# Defining stem cell dynamics and migration during wound healing in mouse skin epidermis

**DOI:** 10.1038/ncomms14684

**Published:** 2017-03-01

**Authors:** Mariaceleste Aragona, Sophie Dekoninck, Steffen Rulands, Sandrine Lenglez, Guilhem Mascré, Benjamin D. Simons, Cédric Blanpain

**Affiliations:** 1 Université Libre de Bruxelles, IRIBHM, Brussels B-1070, Belgium; 2 Cavendish Laboratory, Department of Physics, University of Cambridge, J.J. Thomson Avenue, Cambridge CB3 0HE, UK; 3 The Wellcome Trust/Cancer Research UK Gurdon Institute, University of Cambridge, Tennis Court Road, Cambridge CB2 1QN, UK; 4 Wellcome Trust-Medical Research Council Stem Cell Institute, University of Cambridge, Cambridge CB2 1QR, UK; 5 WELBIO, Université Libre de Bruxelles, Brussels B-1070, Belgium

## Abstract

Wound healing is essential to repair the skin after injury. In the epidermis, distinct stem cells (SCs) populations contribute to wound healing. However, how SCs balance proliferation, differentiation and migration to repair a wound remains poorly understood. Here, we show the cellular and molecular mechanisms that regulate wound healing in mouse tail epidermis. Using a combination of proliferation kinetics experiments and molecular profiling, we identify the gene signatures associated with proliferation, differentiation and migration in different regions surrounding the wound. Functional experiments show that SC proliferation, migration and differentiation can be uncoupled during wound healing. Lineage tracing and quantitative clonal analysis reveal that, following wounding, progenitors divide more rapidly, but conserve their homoeostatic mode of division, leading to their rapid depletion, whereas SCs become active, giving rise to new progenitors that expand and repair the wound. These results have important implications for tissue regeneration, acute and chronic wound disorders.

## Abstract (web summary)

Wound healing is essential to repair the skin after injury and distinct stem cells in the epidermis are known to contribute to the process. Here the authors perform molecular, functional and clonal analysis and reveal the individual contribution of stem cells coming from different epidermal compartments to the wound-healing process in mice.

The skin epidermis is a stratified epithelium that acts as a barrier protecting the animals against infections, trauma and water loss^1^. When the skin barrier is disrupted, a cascade of cellular and molecular events is activated to repair the damage and restore skin integrity. Defects in these events can lead to improper repair causing acute and chronic wound disorders^2^.

Wound healing (WH) is organized in three stages^1^,^2^,^3^,^4^: the inflammation stage starts immediately, and is associated with the formation of the blood clot and the recruitment of inflammatory cells. The second stage is the regenerative phase associated with re-epithelialization of the wound, the creation of new epidermal cells and the formation of the granulation tissue. Finally, the last stage, which can last for months, involves the remodelling of the epidermis, dermis and extracellular matrix (ECM). Different epidermal SCs coming from the hair follicle (HF), isthmus, infundibulum and interfollicular epidermis (IFE) contribute to WH^5^,^6^,^7^,^8^,^9^,^10^,^11^,^12^. However, it remains unclear how different SCs populations can balance proliferation, differentiation and migration during the healing process, and whether they conform to the same proliferative dynamics. It also remains unclear whether these cells simply increase their proliferation rate, maintaining a homoeostatic mode of division, or whether they switch to a proliferative mode of division leading to more symmetrical cell duplication to facilitate the expansion of newly formed skin.

Here, using whole-mount tail epidermis, we identify and characterize molecularly and functionally two spatially distinct epithelial compartments surrounding the wound: a proliferative hub and a migrating leading edge (LE). We define the spatiotemporal dynamics of these two compartments over the re-epithelialization stage. We uncover the molecular signatures associated with these two distinct epidermal compartments and demonstrate that proliferation, migration and differentiation can be uncoupled during the early stage of wound repair. To understand the mode of division and the cellular hierarchy of different populations of epidermal cells, we perform a detailed quantitative clonal analysis and mathematical modelling of the individual behaviour IFE and infundibulum cells during WH. We show that at the beginning of WH, because of the incapacity of progenitors to switch from homoeostatic (asymmetric cell fate outcome at the population level) to a proliferative (symmetric renewal) mode of division, the important increase in cell proliferation leads to minimal tissue regeneration with a massive loss of progenitors through differentiation. As SCs become activated, they undergo rapid asymmetric cell fate outcome generating new SCs and progenitors that promote tissue expansion, visible as streaks of cells spanning from the proliferative hub to the centre of the wound. This clonal dynamic is very similar for different populations of epidermal SCs coming from different skin regions, suggesting that this cellular behaviour helps to maximize the regenerative process.

## Results

### Spatiotemporal proliferation and migration during WH

**Figure 1:**
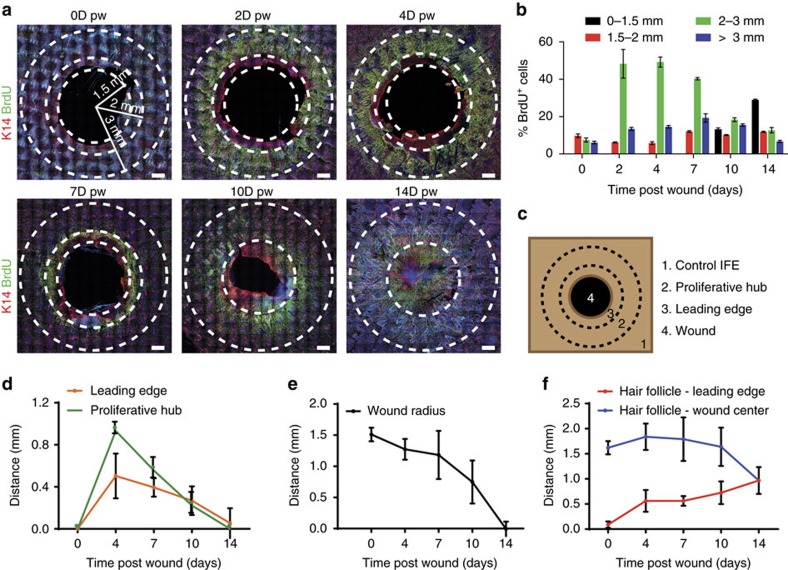
Respective contribution of cell proliferation and migration during WH. (**a**) Representative immunostaining of K14 (red) and BrdU (green) in whole-mount skin epidermis of the wounded region at the different time points. Dashed lines limit the wounded area, the LE and the proliferative hub. Scale bar, 500 μm. (**b**) Quantification of the percentage of BrdU positive cells according to the distance from the wound centre (*n*=5,000 cells/region counted from three different mice). (**c**) Descriptive scheme showing the situation in the early days after wound and the localization of the two different areas around the wound between 2 and 7 days after wound. (**d**) Measure of the width of the LE (orange line) and the proliferative hub (green line) overtime. Five measures were taken per wound (*n*=3 mice). (**e**) Measure of the average wound radius overtime. Five different measures were taken per wound (*n*=3 mice). (**f**) Measure of the distance between the nearest HF and the LE (red line) and the distance between the HF and the wound centre (blue line). Five different measures were taken per wound (*n*=3 mice).

To define the role of cell proliferation during the regenerative stage of WH, we performed a 3 mm punch biopsy in the tail skin of adult mice and analysed the result of short-term BrdU incorporation by confocal microscopy on whole-mount epidermis at different time points during WH (Fig. 1a). Immediately after wounding, there was no increase in BrdU incorporation. However, at day 2 (D2) and even more at D4 following wounding, we found that BrdU incorporation was increased by 5-fold in a zone spanning from 500 μm to 1.5 mm from the LE, with 40% of basal cells entering into cycle during a period of 4 h (Fig. 1b). The width of the annulus of cells that proliferated around the wound progressively decreased with time (Fig. 1a,c,d). We found that epidermal cells at the LE, spanning a distance of 500 μm from the wound front, did not incorporate BrdU at any time point from D2 to D7 following wounding (Fig. 1a–c). This showed that cells at the LE of the epidermal sheath, which ensures skin regeneration, do not proliferate actively, but migrate to the centre of the wound. These results confirm the existence of a migrating LE that has been hypothesized for several decades based on the histological examination of wounded tissues and *ex-vivo* skin explants^3^. Reaching a maximal size at D4 following wounding, the size of the non-proliferating LE zone progressively decreased over time, suggesting that the specification and differentiation of LE cells occurs only during the early stage of WH (Fig. 1a,d). After D14, the wound edges fused at midline and proliferation resumed at the centre of the wound region (Fig. 1a,e).

As wound contraction contributes to wound closure^13^, we assessed the relative importance of epidermal regeneration and wound contraction to the overall wound repair. As *de novo* HF formation only occurs with more extensive wounding and at a later stage^14^, wound contraction was measured by the distance between the HF and the wound centre at D0 minus the same measurement at a given time point, while the newly formed epidermis was measured by the difference between the radius at D0 (1.5 mm) and the radius at any time point minus the contraction. Surprisingly, we found that the distance between HF triplets and the centre of the wound after the punch biopsy did not decrease significantly from D0 to D7, where proliferation was maximum, suggesting that proliferation is not very productive during the initial stage of wound repair (Fig. 1e,f). From D10 to D14, this distance decreased linearly in time until re-epithelialization was completed (Fig. 1e,f). At this stage the average distance between the HF triplets and the wound centre is 0.9 mm, suggesting that an epithelial regeneration contributes approximately to two-thirds of the healing process, and wound contraction (0.6 mm) is responsible for the remainder.

### Cell shape and polarity during WH

**Figure 2:**
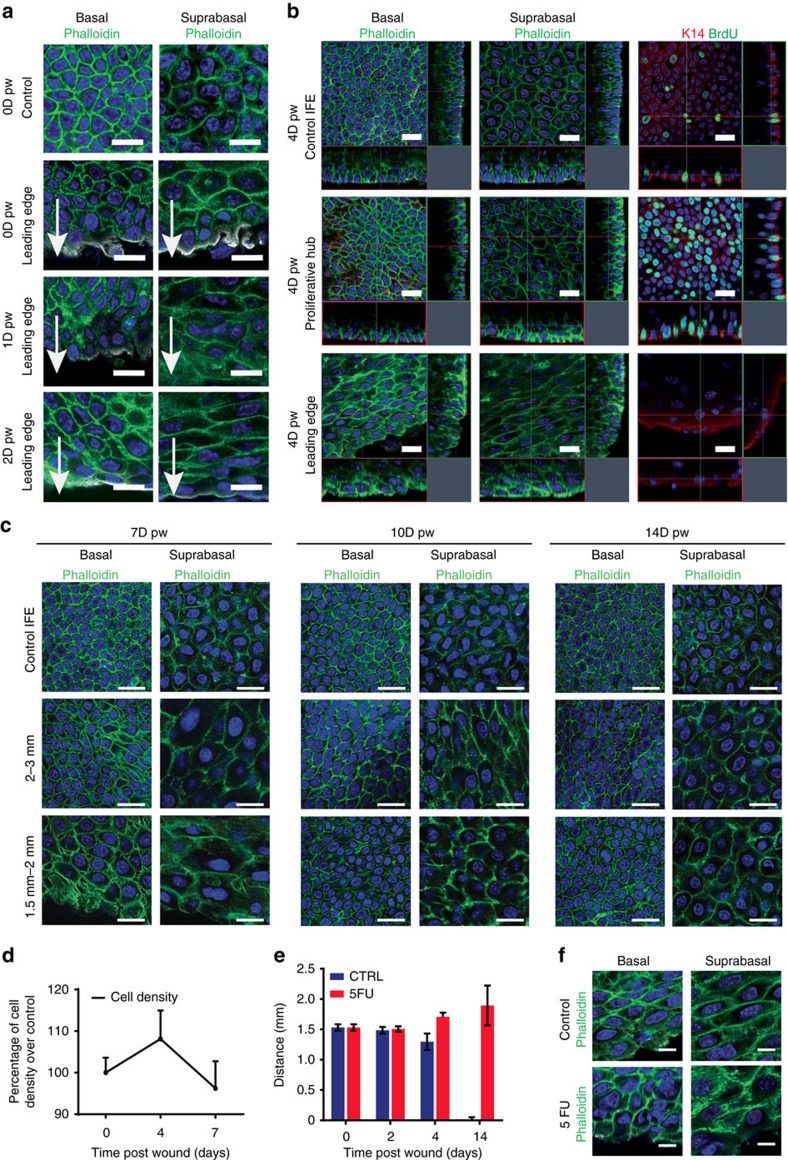
Modifications of cell polarity and cell shape during WH. (**a**) Representative confocal analysis of whole-mount epidermis stained for F-actin with phalloidin (green) and β4-integrin (white) showing the different shape of basal (left) and suprabasal (right) cells at the LE 0, 1 and 2 days after wound compare to a control area. Arrows indicate the direction of the wound. Scale bar, 20 μm. (**b**) Left and middle panels: representative confocal analysis of whole-mounted epidermis stained for F-actin with phalloidin (green) and β4-integrin (white) showing the shape of the basal (left) and suprabasal (middle) cells in the different regions, 4 days after wound. Right panel: immunostaining for BrdU (green) and K14 (red) in the different regions 4 days after wound. (**c**) Representative confocal pictures of whole-mounted epidermis immunostained for F-actin with phalloidin (green) showing the shape of the basal and suprabasal keratinocytes in the control area, proliferative hub (2–3 mm) and LE (0–2 mm) 7, 10 and 14 days after wound. Nuclei are stained with Hoechst (blue). Scale bar, 20 μm. (**d**) Percentage of cell density at 0, 4 and 7 days post wound in the proliferative hub normalized by a control area. Five different measures were taken per wound (*n*=4 mice). (**e**) Measure of the wound radius after 5-FU topical treatment compared to control-untreated mice (*n*=3 mice). (**f**) Representative confocal pictures of whole-mounted epidermis stained for F-actin with phalloidin (green) showing the elongated cells at the LE in the untreated mice and the random orientation of the cells in the same area after 5-FU treatment. Nuclei are stained with Hoechst (blue). Scale bar, 20 μm. Wound centre is at the bottom edge of the pictures.

The shape and size of the epidermal cells, which is the reflection of the forces that epidermal cells experience during the regeneration process, was very different depending on the wound region and the time point following wounding (Fig. 2a–c). At D0, the basal cells of the LE appeared less compacted (Fig. 2a), consistent with a relaxation in the force exerted on the wound edge. At D1, the LE cells were elongated toward the wound centre (Fig. 2a), as previously shown^15^, consistent with the active migration of the LE cells toward this point. At D4, basal cells far from the wound presented a regular cuboidal/hexagonal shape (Fig. 2b). The density of basal cells in the proliferative zone was increased, leading to a more compressed cell shape (Fig 2b,d). In contrast, in the non-proliferative zone, the basal cells were bigger, polarized in the same direction, and elongated along an axis perpendicular to the direction of the wound closure (Fig. 2b). This suggests that, at D4 and thereafter, the movement of the LE is a passive process possibly mediated by the proliferating cells (Fig. 2b). Consistent with this notion, blocking epidermal cell proliferation by 5-fluorouracil (5-FU), which inhibited the re-epithelialization process and WH (Fig. 2e), prevented the perpendicular polarization of the LE at D4 (Fig. 2f). These data demonstrate that the two distinct epidermal compartments, the proliferative hub and the LE, present different cell shape and polarity that change with time, likely reflecting the difference in the physical forces that these different zones experience at the different stages of WH.

### Molecular signature of LE and proliferation hub during WH

**Figure 3:**
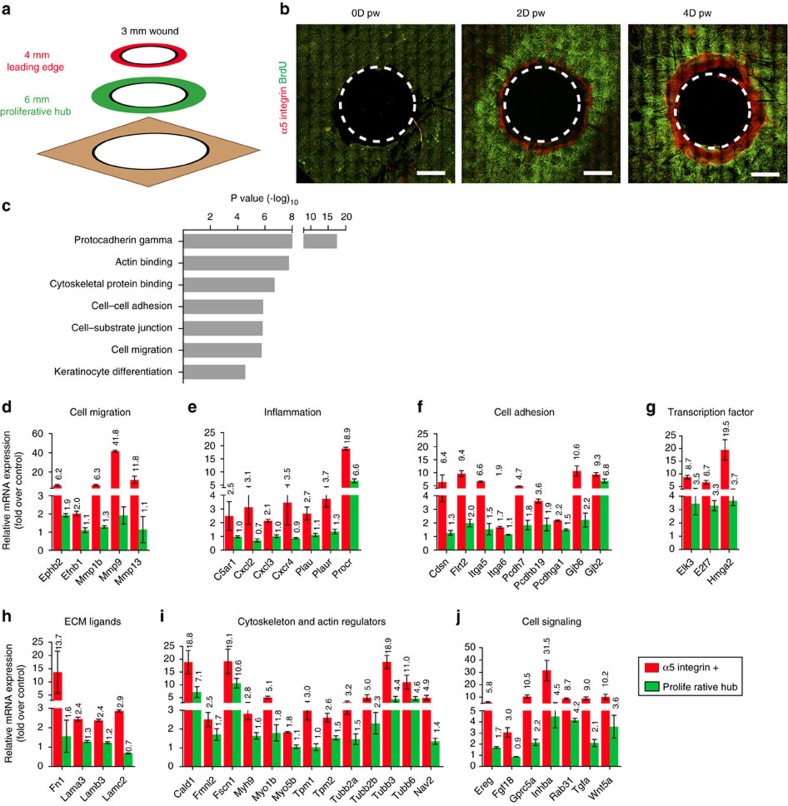
Molecular signature of the proliferative hub and the LE during WH. (**a**) Scheme showing the strategy used to isolate the LE with a 4 mm punch biopsy and the proliferative hub with a 6-mm-punch biopsy. (**b**) Representative maximum intensity projection of confocal pictures showing the immunostaining of whole-mount epidermis with α5-integrin (red) and anti-BrdU (green) 0, 2 and 4 days after wound. Note the expression of the α5-integrin by the non-proliferative LE cells only. (**c**) Gene ontology enrichment in the LE 4 days after wound (*n*=3). (**d**–**j**) LE signature. List of genes upregulated in the α5-integrin positive cells of the LE compared with the proliferative hub (*n*=3). These genes are implicated in cell migration (**d**), inflammation (**e**), cell adhesion (**f**), transcription (**g**), ECM composition (**h**), cytoskeleton and actin regulators (**i**) and cell signalling (**j**).

To define the molecular features associated with the formation of the proliferative hub and the LE, we performed transcriptional profiling of different concentric rings of the wound using different sizes of punch biopsy and fluorescence-activated cell sorting (FACS) sorting. A first punch biopsy of 4 mm in diameter around the wound was used to enrich for LE cells (spanning two times 500 μm, the average width of the LE), and a second punch biopsy (6 mm) was enriched for the proliferative hub of the wound (Fig. 3a; Supplementary Fig. 1). We performed a third biopsy far from the wound corresponding to control normal unwounded epidermis. We performed duplicate microarrays of these three skin regions at D4 and D7 post-wounding. We found genes upregulated in both wound regions at the two different time points, which correspond to a generic wound-healing signature. This gene signature included genes regulating cell adhesion (for example, *Dsc2*), cytoskeleton (for example, *Krt6*, *Krt17*), inflammation (for example, *Il24*, *Il33 S100a8/a9*), cell signalling (for example, *Areg*, *Ereg*, *Emb*, *Epgn*), (Supplementary Fig. 2a) and cell cycle-related genes (for example, *Ccna2*, *Ccnb1*) (Supplementary Fig. 2b). For some genes, such as *Krt6* (refs 3, 16, 17, 18, 19), in which expression was confirmed by immunofluorescence (Supplementary Fig. 2c), *Il24*, *S100a8/a9* or the EGFR ligands, their role in the regulation of WH has previously been described^20^,^21^,^22^,^23^. In other cases, including *Fscn1*, *Emb*, *Sprr1b and Sprr2h*, genes were not known to be involved in skin WH.

We next defined which genes were preferentially upregulated and downregulated in the LE as compared with the proliferative hub (the LE signature) (Supplementary Fig. 2d–f). We found that *α5-integrin* was highly enriched in the LE signature (Supplementary Fig. 2e); consistent with a previous study that showed that *α5-integrin* was expressed at the LE of human skin explants *ex vivo*^24^,^25^,^26^,^27^ and at the LE during eyelid closure, a developmental process that involves epidermal cell migration^28^, reminiscent of the LE during WH. Whole-mount immunostaining confirmed the rapid upregulation of α5-integrin in the non-proliferative cells of the LE *in vivo* (Fig. 3b). To refine the molecular signature of the LE without contamination of proliferative cells, we isolated α5-integrin positive cells from a 4 mm punch biopsy by FACS at D4 following wounding (Supplementary Fig. 3) and performed microarray analysis in triplicates. These molecular analyses confirmed the preferential expression of many of the previously described genes expressed during WH, validating the approach used here and allowing for the first time to distinguish the spatial localization of these genes at the LE and/or in the proliferative hub. In addition, the gene signatures of the proliferative and LE cells during wounding uncover many novel genes not previously described during WH and tissue regeneration (Fig. 3c–j). Gene Ontology Enrichment (GO) analysis revealed that the genes upregulated in the LE comprised genes regulating cell adhesion, cytoskeleton organization, epidermal differentiation, cell migration and other processes involved in WH (Fig. 3c). The most upregulated genes of the LE signature were genes coding for proteins regulating cell migration including several metalloproteinases (MMPs) (*Mmp9*, *Mmp13*, *Mmp1b*) (Fig. 3d), whereas *Timp3*, an inhibitor of metalloproteinase, was the most downregulated gene (Supplementary Fig. 2f), suggesting that the high level of MMPs expressed by the cells of the LE promote the remodelling of the ECM at the wound front allowing the front cells to progress toward the centre. MMPs also help the breakdown of the hemi desmosomes that anchor the cells at the basal membrane and are therefore essential for the movement of basal cells. MMPs deletion in flies and mice results in wound-healing defects due to defective cell elongation, cytoskeleton and basal membrane remodelling as well as cell migration^29^,^30^,^31^,^32^,^33^. The migrating zone also expressed high level of *urokinase* (*Plau*) and *plasminogen activator* (*Plaur*) (Fig. 3e), two key fibrinolytic proteins contributing to the remodelling of the blood clot during WH^34^. The LE also expressed high level of *Ephb2* and *Efnb1* (Fig. 3d), a receptor and its ligand, which have recently been shown to control WH^35^ as well as other genes such as *Cxcr4*, *C5ar1*, *Myh9*, *Procr*, *Wnt5a*, *Elk3* (Fig. 3e,g,i,j), which regulate cell migration in other cellular contexts. We found that *Inhibin-βa*, a subunit of Activin A was overexpressed preferentially at the LE (Fig. 3j), in good accordance with in-situ hybridization of *Inhibin-βa* during WH^36^ and the previously reported role of Activin A during WH in mice^37^,^38^,^39^,^40^,^41^,^42^.

Our LE signature encompassed many genes controlling cell adhesion, including several protocadherins (*Pcdh7*, *Pcdhb19*, *Pcdhga1*) (Fig. 3f), integrins (*Itga5*, *Itga6*) (Fig. 3f) and some of their ECM ligands (*Fn1*, *Lama3*, *Lamb3*, *Lamc2*) (Fig. 3h), desmosomes (*Cdsn*)^43^ and gap junction proteins (*Gjb6/Cx30* and *Gjb2/Cx26*) (Fig. 3f). Corneodesmosin (*Cdsn*), a desmosome protein of the wound edge signature (Fig. 3f) was reported to be expressed using transgenic reporter mice in the wound edge and in the inner root sheath (IRS) of the hair^44^.

Many genes controlling cell cytoskeleton and actin remodelling including actin regulators (*Fscn1*, *Cald1*, *Nav2*, *Fmnl2*), myosin (*Myo1b*, *Myo5b*, *Myh9*, *Tpm1*, *Tpm2*) and tubulin (*Tubb2a*, *Tubb3*, *Tubb6*), were preferentially overexpressed at the LE (Fig. 3i), and *Tubb6* was previously shown to be upregulated during wounding^19^. These genes may control the morphology, polarity, rigidity of the cell cortex and migration during WH.

Several genes that might regulate the quiescence of the LE surfaced from the microarray analysis. *Gprc5a*, an orphan G protein coupled receptor, acting as tumour suppressor genes in the lung by negatively regulating EGFR and Stat3 signalling^45^,^46^,^47^, was strongly upregulated at the LE (Fig. 3j.). The upregulation of *E2f7* (ref. 48) or *Fgf18* (ref. 49), genes that promote cell quiescence in other contexts may also contribute to shut down of proliferation in the wound LE (Fig. 3g,j).

### Spatiotemporal expression of the LE signature during WH

**Figure 4:**
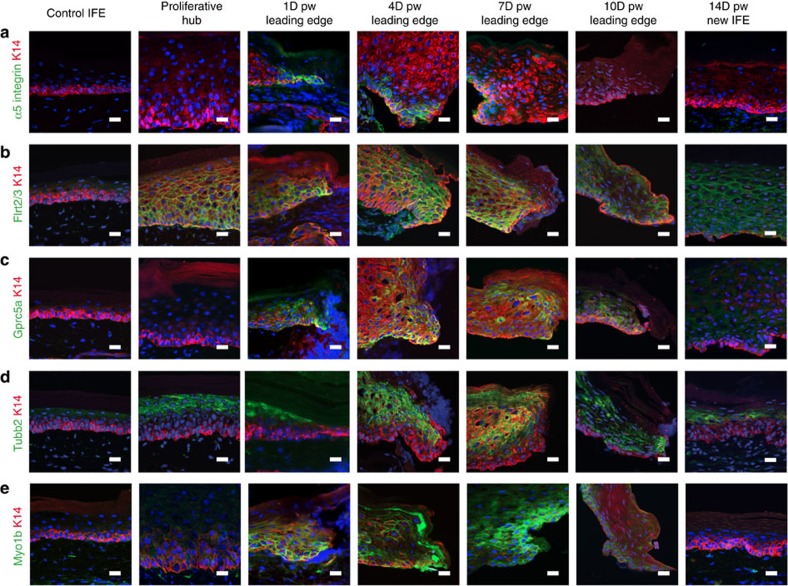
Spatiotemporal expression of the LE signature during WH. (**a**) Representative immunofluorescence of α5-integrin (green) on skin section showing a strong and transient expression of Itgα5 in the basal cells of the LE at D1, D4 and D7 after wound. (**b**) Representative immunofluorescence of Flrt2/3(green) on skin section showing its expression in the proliferative hub and the LE in basal and suprabasal cells from 1 to 7 days after wound and progressively decreasing 10 and 14 days after wound. (**c**) Representative immunofluorescence of Gprc5a (green) on skin sections showing its overexpression in the basal and suprabasal cells at the LE at the different time points. (**d**) Representative immunofluorescence of Tubb2 (green) showing its higher expression in the suprabasal cells both in the proliferative hub and the LE after wound. (**e**) Representative immunofluorescence for Myo1b (green) showing positive signal in the cells of the LE from D1 to D7. In all the pictures from **a**–**e** K14 is in red and the nuclei are stained with Hoechst (blue). Scale bar, 20 μm.

Immunostaining performed against several of these newly identified markers of the LE signature including cell adhesion, receptor and cytoskeleton proteins (*Flrt2*, *Gprc5a*, *Tubb2*, *Myo1b*, *Itga5*) confirmed their preferential enrichment at the LE of the wound as predicted by our microarray analysis (Fig. 4a–e). Itga5 was expressed preferentially in the basal cells of the LE (Fig. 4a). Flrt2, a repulsive guidance protein that regulates the migration of neuronal progenitors during embryonic development^50^ was more expressed in the cells of the wound edge than in the proliferation zone, but was not present in the normal skin epidermis (Fig. 4b). Gprc5a was expressed at the LE of the wound at D4 (Fig. 4c), and similarly to Cdsn^44^, Flrt2 or Tubb2, to the IRS or precortex cells of the HFs (Fig. 4b–d; Supplementary Fig. 4a–c). Myosin 1b, a tension-sensitive myosin was also expressed in all cell types of the LE (Fig. 4e), which by regulating actin foci stability, controls cell migration or repulsion. Myosin 1b was also expressed in the bulge and outer root sheath (ORS) of the HFs (Supplementary Fig. 4d). The expression of all these newly identified molecular markers of the wound LE signature decreased overtime and at D14 post-wound, when the opposite margin of epidermal cells fused together, these markers were not expressed anymore (Fig. 4a–e). These data demonstrate the transient nature of this wound LE structure.

### Uncoupling proliferation and differentiation during WH

**Figure 5:**
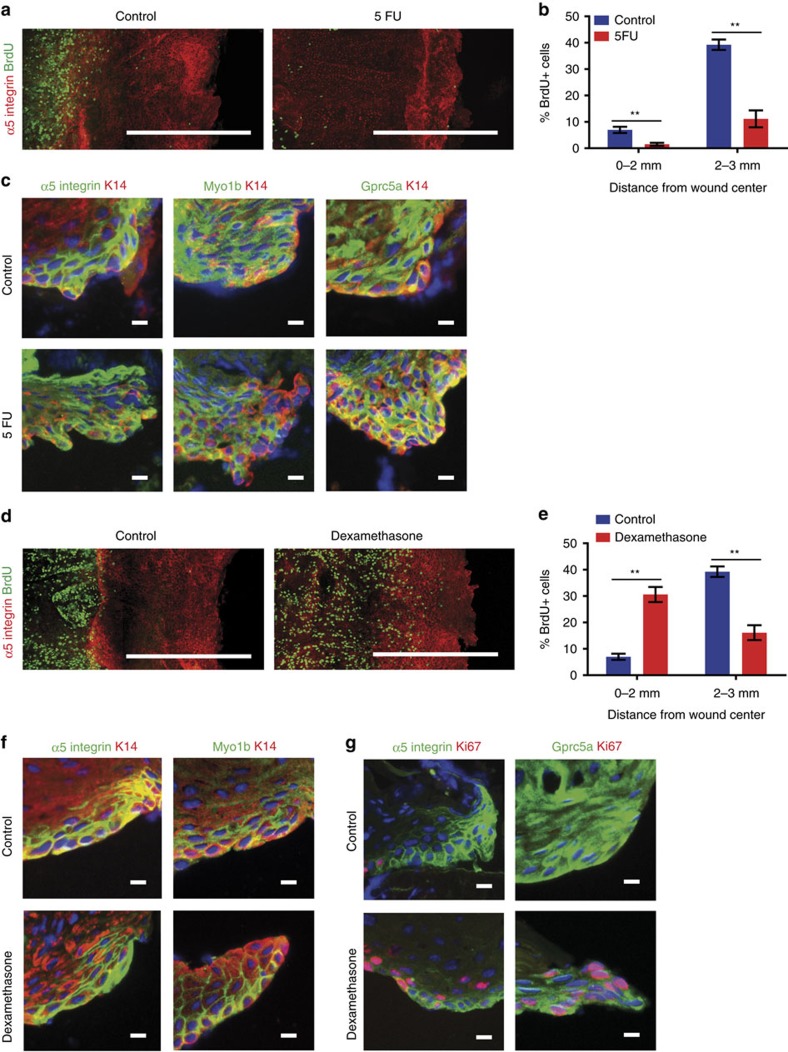
Mechanisms regulating the formation of the LE during WH. (**a**) Maximum intensity projection of representative confocal pictures of untreated (control) and 5-FU-treated mice (right). Scale bar, 500 μm. (**b**) Quantification of the percentage of BrdU positive cells in the LE (0–2 mm) and the proliferative hub (2–3 mm) of untreated (control) and 5-FU-treated mice (**P=0.0079 by Mann–Whitney test, *n*=5 mice). (**c**) Representative immunostaining of skin sections showing the presence of the α5-integrin, Myo1b and Gprc5a (green), and K14 (red) at the LE in normal wound and after 5-FU treatment. Scale bar, 10 μm. (**d**) Maximum intensity projection of representative confocal pictures showing whole-mounted epidermis stained for α5-integrin (red) and BrdU (green) 4 days after wound under normal condition (left) and after anti-inflammatory treatment with dexamethasone (right). Scale bar, 500 μm. (**e**) Quantification of the percentage of BrdU positive cells in the LE (0–2 mm) and the proliferative hub (2–3 mm) of untreated and dexamethasone-treated mice (**P= 0.0079 by Mann–Whitney test, *n*=5 mice). (**f**,**g**) Representative immunostaining on skin sections showing the expression of the α5-integrin, Myo1b (green) and K14 (red) (**f**), and the expression of α5-integrin, Gprc5a (green) and Ki67 (red) (**g**) at the LE in untreated and dexamethasone-treated mice. Scale bar, 10 μm.

To gain further insights into the mechanisms that specify these two distinct regions, we determined whether the fate and the differentiation programme of the LE are linked to cell division, by assessing the impact of blocking cell proliferation on the fate of LE cells. Topical application of 5-FU, which strongly decreased epidermal cell proliferation, did not prevent or impair the expression of LE markers (Fig. 5a–c), demonstrating that the particular differentiation programme of the LE is specified independently of cell division.

As inflammation plays an important role in orchestrating the early step of WH^1^,^2^,^51^, we assessed the impact of blocking inflammation on these two distinct epidermal compartments during re-epithelialization. Interestingly, treating the wounded mice with dexamethasone, a potent anti-inflammatory drug, reactivate proliferation in the LE without impairing its particular gene expression signature (Fig. 5d–g) demonstrating that, the LE-specific gene signature is not associated with terminal differentiation (Fig. 5f,g). These data show that glucocorticoid treatment suppresses a negative regulator of cell cycle acting on the LE. As glucocorticoid can also directly act on keratinocytes^52^, this negative regulator may originate either from the keratinocytes or from the inflammatory cells and the granulation tissue. Although, these data do not allow to discriminate whether the inhibition of proliferation at the LE is regulated by an intrinsic or an extrinsic mechanism, these results provide compelling evidence that the LE-specific cellular quiescence and gene expression signature can be functionally and molecularly uncoupled.

### Clonal analysis of IFE SC during WH

**Figure 6:**
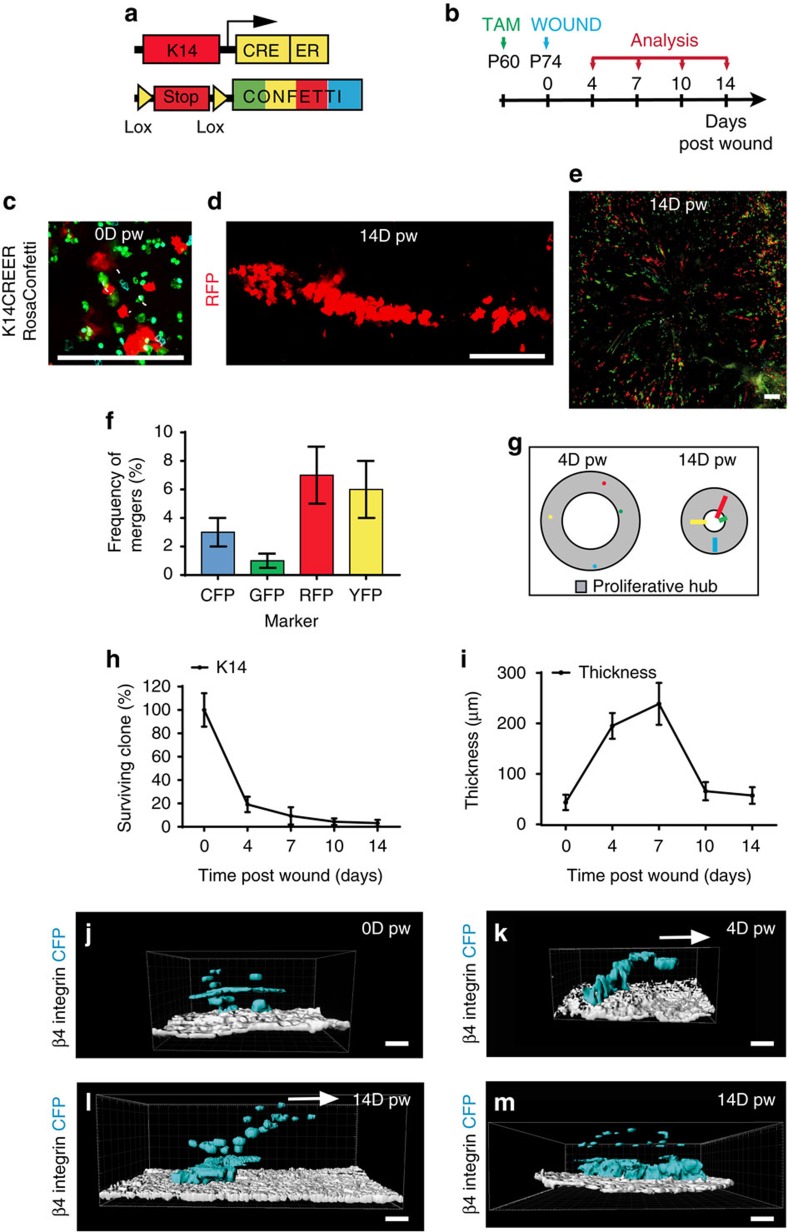
Clonal analysis of IFE SC and progenitors during WH. (**a**) Genetic labelling strategy used to trace K14 IFE progenitors during WH. (**b**) Time line of the wound experiment. *K14CREER/RosaConfetti* mice were induced with Tamoxifen at 2 months of age and wounded 2 weeks after. The samples were collected 0, 4, 7, 10 and 14 days after wound. (**c**,**d**) Maximum intensity projection of representative confocal pictures showing K14CREER clones 0 days (**c**) and 14 days (**d**) after wound. (**e**) Maximum intensity projection of a representative confocal picture showing whole-mounted wounded epidermis from *K14CREER RosaConfetti* 14 days after wound. Scale bar, 500 μm. (**f**) Frequency of mergers calculated for each confetti colour. (**g**) Representative scheme showing the position of the clones (coloured dots) in the proliferative hub (grey area) 4 days after wound and the streaks they form 14 days after wound (coloured lines). (**h**) Quantification of the percentage of K14CREER surviving clones overtime after wound showing the massive loss of basally attached clones between day 0 and day 4 post wound. (**i**) Measure of the thickness of the epidermis in the proliferative hub 0, 4, 7, 10 and 14 days after wound. (**j**–**m**) 3D reconstruction using Imaris software of representative *K14CREER RosaConfetti* CFP clones imaged by confocal microscopy during WH. To identify the basal cells attached to the basal lamina, samples were stained for b4 integrin (white). In the control unwounded area (**j**), suprabasal cells are found on the top of basal cells. The picture shows a clone composed of 11 cells, 4 basal and 7 suprabasal cells. In contrast, during healing, the newly produced suprabasal cells are migrating on the top of basal cells toward the wound centre (on the right) (**k**–**m**). In **k**, the clone has the same size as the control (11 cells) but has more suprabasal cells (9 suprabasal and only 2 basal cells). The clone at D14 shown in **l** has expanded to reach 39 cells in total (4 of them are basal cells). In **m**, another clone by contrast expanded basally (83 total cell size, 33 basal cells) at D14 post wound. Arrows indicate cell movement. Scale bar, 20 μm.

To follow the progeny of the basal epidermal cells, and study their cellular dynamics at the single cell level, we performed clonal analysis on *K14CREER/Rosa Confetti* mice targeting preferentially IFE SCs^12^ (Fig. 6a). We administrated Tamoxifen (TAM) 14 days before wounding and analysed the respective clonal contribution during the healing process (Fig. 6b). At the end of the re-epithelialization, K14-labelled cells gave rise to long streaks of labelled cells directed toward the LE of the wound (Fig. 6c–e). With a clone merger probability estimated at roughly 5% (Fig. 6f; Supplementary Note 1), we deduced that streaks labelled with the same fluorescent protein were clonal in origin, derived from single SCs. The clonal lines were often interrupted by unlabelled cells (Fig. 6d,e), suggesting that a cycle of active SC proliferation followed by cell intercalation from neighbouring clones (or clonal fragmentation) occurs repetitively during WH. Interestingly, all of these fragmented clonal streaks originate from the proliferative hub previously described (Fig. 6g). Quantification of the clonal persistence revealed that more than 90% of IFE-labelled clones were lost during the first week following wounding (Fig. 6h), consistent with the majority of progenitors maintaining homoeostatic behaviour, leading to a progressive decrease in the labelled cell fraction^12^,^53^,^54^. Importantly, this clonal dynamic contrasts with that reported for oesophagus, where repair seems to involve a switch of progenitors to a proliferative mode of division^55^, or following *in vitro* culture of human keratinocytes^56^. Consistent with a major increase in the rate of terminal differentiation, as measured by clonal persistence, the epidermal thickness increased during the same period (Fig. 6i).

Three-dimensional analysis of labelled clones revealed that, in contrast to clones in the control regions that are composed of stacks of cells that lie on the top of each other (Fig. 6j), at D4 K14CREER IFE SCs gave rise to basal and suprabasal cells that migrate toward the wound edge (Fig. 6k). The restriction of the basal footprint of the clone at the trailing edge suggest that marked SCs undergo predominantly asymmetric cell division, giving rise to a steady production of progenitors. Interestingly, despite the high rate of proliferation observed in the first days following wounding, at this time point, K14CREER clones are not on average bigger as compared to D0, suggesting that the effect of SC renewal is not yet observed at the population level.

By D14, most of the persisting K14CREER clones became enlarged in their basal attachment and were composed of a majority of suprabasal cells (Fig. 6l), while others formed long streaks of basal cells and suprabasal cells (Fig. 6m), suggesting that these clones produced an increased number of basal progenitors leading to basal cell expansion. Interestingly, these streaks originate from a minority (>1%) of K14CREER-induced cells and seem to be responsible for only part of the wound regeneration (for details see Supplementary Note 1).

It has been proposed that, during wounding, differentiated suprabasal cells can revert back to a progenitor state and actively contribute to repair^57^,^58^. To test this possibility, we administrated TAM to *InvCREER/RosaYFP* that labelled suprabasal cells and rare basal cells (Supplementary Fig. 5a,b), performed a punch biopsy and analysed the contribution of labelled suprabasal cells during wounding. Ten days after wounding, most of the lineage labelled suprabasal cells had been shed from the skin surface or remained present as spinous or granular cells. Despite the high frequency of suprabasal cell labelling, we found only very rare basal cells initially targeted by the InvCREER, and these contribute minimally and transiently to the wound repair as previously described^3^. We found no evidence that suprabasal cells can revert back to a progenitor-like state, as the density of basal cells contributing to the wound repair is much lower compared to that of basal cells labelled at induction (Supplementary Fig. 5a,b). These data suggest that dedifferentiation of differentiated suprabasal cells does not contribute to WH in the tail epidermis.

### Clonal analysis of infundibulum SC during WH

**Figure 7:**
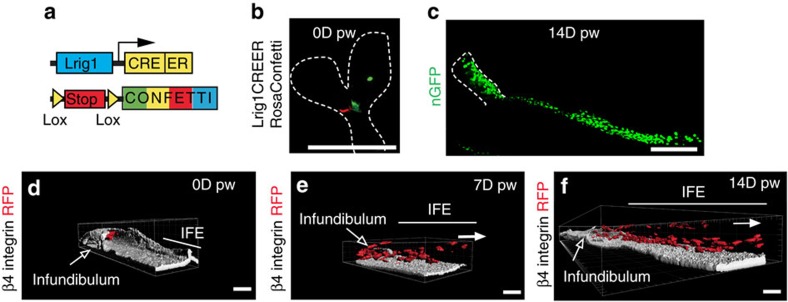
Clonal analysis of infundibulum SC during WH. (**a**) Genetic labelling strategy used to trace Lrig1 upper HF cells during WH. (**b**,**c**) Maximum intensity projection of representative confocal pictures showing Lrig1CREER clones 0 days (**b**) and 14 days (**c**) after wound. (**d**–**f**) 3D reconstruction of RFP (red) positive Lrig1-targeted clones with Imaris software. The β4-integrin (white) is marking the basal side. Initially and in normal conditions, Lrig1-targeted clone is confined in the upper part of the HF/infundibulum (**d**). Upon wound, the progeny of the clone is moving outside of the HF/infundibulum and lines of cells can be seen in the IFE 7 days (**e**) and 14 days after wound (**f**). The white arrows show the direction of the wound. Scale bar, 50 μm.

To assess whether different types of SCs arising from distinct epidermal regions present different clonal dynamics during WH, we performed clonal analysis on *Lrig1CREER/Rosa Confetti* mice targeting the upper HF SCs that include cells from the infundibulum, junctional zone and sebaceous gland, and that have been shown to contribute to WH^8^ (Fig. 7a,b). At D14, most of the Lrig1-labelled cells give rise to long streaks of progeny from the infundibulum to the LE (Fig. 7c) with the clones presenting the same fragmentation reported for the K14 tracing (Figs 6d,e and 7c). The 3D reconstruction showed that the Lrig1 clones, starting from D7, had an analogous cellular composition as the K14 with long streak of basal and suprabasal cells emanating from the infundibulum and directed toward the wound centre (Fig. 7d–f).

### Similar clonal dynamic of different epidermal SCs during WH

**Figure 8:**
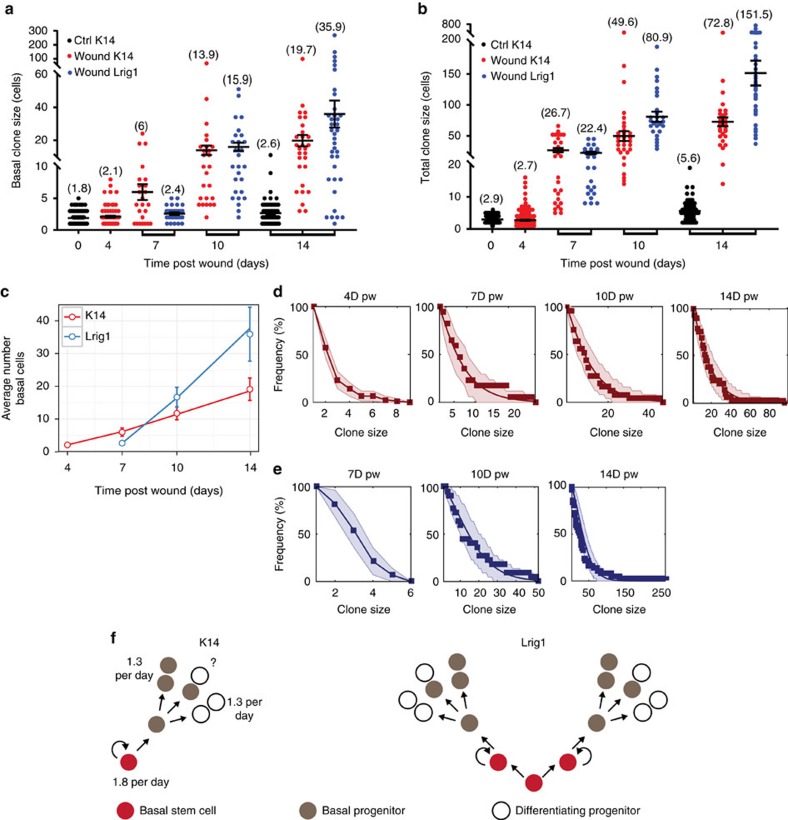
Distinct epidermal stem cells present similar clonal dynamic during WH. (**a**,**b**) Clone size distribution showing the number of basal cells (**a**) and the total number of cells (**b**) per clone counted in *K14CREER* and *Lrig1CREER RosaConfetti* mice. (**c**) Average number of basal cells per clone in K14CREER (red) and Lrig1CREER (blue) clones. Solid lines are model predictions. The linear growth of both populations suggests that the stem cells fate outcomes are balanced but Lrig1 SCs achieved at the beginning one more symmetric division initially than K14 SCs. (**d**,**e**) Cumulative frequencies of K14CREER (**d**) and Lrig1CREER (**e**) basal clone size at different days after wound (dots). Solid lines give the model prediction and shaded areas denote the uncertainty of the model given the experimental data (95% confidence intervals). (**f**) Model of SC and progenitors cell fate outcome in IFE for K14 (left) and Lrig1 (right) populations during WH. K14-targeted SCs (red, left panel) divide asymmetrically 1.8 (+0.6; −0.3) times per day and give rise to progenitors (grey, left panel) that divide 1.3 (+1.1; −0.6) times per day which then give rise to differentiated cells following a balanced cell fate outcome. Lrig1-targeted SCs (red, right panel) achieve initially one symmetrical division and then asymmetrical divisions giving rise to progenitors (left grey) which follows the same cell fate outcome as described for K14 SCs.

To gain further insight into the clonal dynamics of the IFE and the HF-derived SCs populations, we used a previously validated biostatistical framework^59^,^60^,^61^ to infer with high confidence the number of cells and cellular composition of clones (basal versus suprabasal cells) arising from single *K14* and *Lrig1CREER/Rosa Confetti*-targeted cells (Fig. 8a–e; Supplementary Note 1). To define how SCs balance proliferation and differentiation in the proliferative hub surrounding the wound edge, we analysed the clonal composition of *K14* and *Lrig1CREER/Rosa Confetti*-derived clones from D0 to D14 after wounding. From D4 to D14, the basal size of clones of both K14 and Lrig1CREER grew remarkably linearly (Fig. 8a,c). At first sight, this behaviour could indicate neutral competition, as has been reported for uninjured epidermis^12^,^62^. However, adapting the standard model of balanced stochastic progenitor cell fate, the observed rate of increase in the average basal size would translate to an unreasonably fast cell cycle time of >6 h. Rather, in this case, the linear increase indicates the labelling of an asymmetrically dividing subpopulation that drives the basal expansion through the steady production of progenitors (Supplementary Note 1). On the basis of the quantitative clonal analysis, we find that the data are consistent with a proliferative hierarchical model in which, during WH, a putative SC population at the apex divide perfectly asymmetrically giving rise to self-renewing progenitors in which the frequency of symmetrical duplication is balanced by symmetrical differentiation (Fig. 8f). Indeed, the existence of a persistent (SC) and a transient (progenitor) basal subpopulation might explain the fragmentation of clonal streaks. From the modelling of the clonal dynamics, we found that such model indeed predicts not only the increase in average basal clone size (Fig. 8c) but also recapitulates the detailed distributions of basal clones sizes in the K14CREER assay (Fig. 8d; Supplementary Note 1). Notably, such a hierarchical model coincides with that inferred from the study of homoeostatic turnover of interscale epidermis, but where the proliferation rate of SCs and progenitors have been massively increased^54^. Surprisingly, the conserved linearity of the average basal clone size and the shape of the size distribution suggested that Lrig1CREER targets SCs belonging to the same hierarchy as that targeted by K14CREER, but where a burst of proliferative activity at the earliest stages of regeneration expands the average number of SCs in each clone (Fig. 8c–f; Supplementary Note 1). Altogether, these data suggest that, irrespective of the epidermal origin, the regenerative stage of WH involves a sustained increase in proliferative activity of a minority of SCs, while keeping the fate behaviour and lineage relationship of SCs and progenitors largely unperturbed from their homoeostatic dependences.

## Discussion

Our study uncovers the clonal dynamics and individual contribution of SCs coming from different epidermal compartments during skin WH in mice. In contrast to what has been proposed for oesophagus repair and the growth of human keratinocytes *in vitro*^55^,^56^, our data show that WH does not increase the self-renewal capacities of progenitors, but rather leads to their massive depletion as proliferation increases. The repair of the skin epidermis does not induce a change in the cellular hierarchy of SCs and progenitors, or a change in the balance between renewal and differentiation but rather involves an increase in the proliferation rate of a small population of SCs that gives rise to progenitors upon asymmetric division leading to a linear increase in the individual clone size over time. Interestingly, IFE and infundibulum SCs present very similar clonal dynamics during wound repair, despite the fact that they are recruited from different regions of the epidermis.

**Figure 9:**
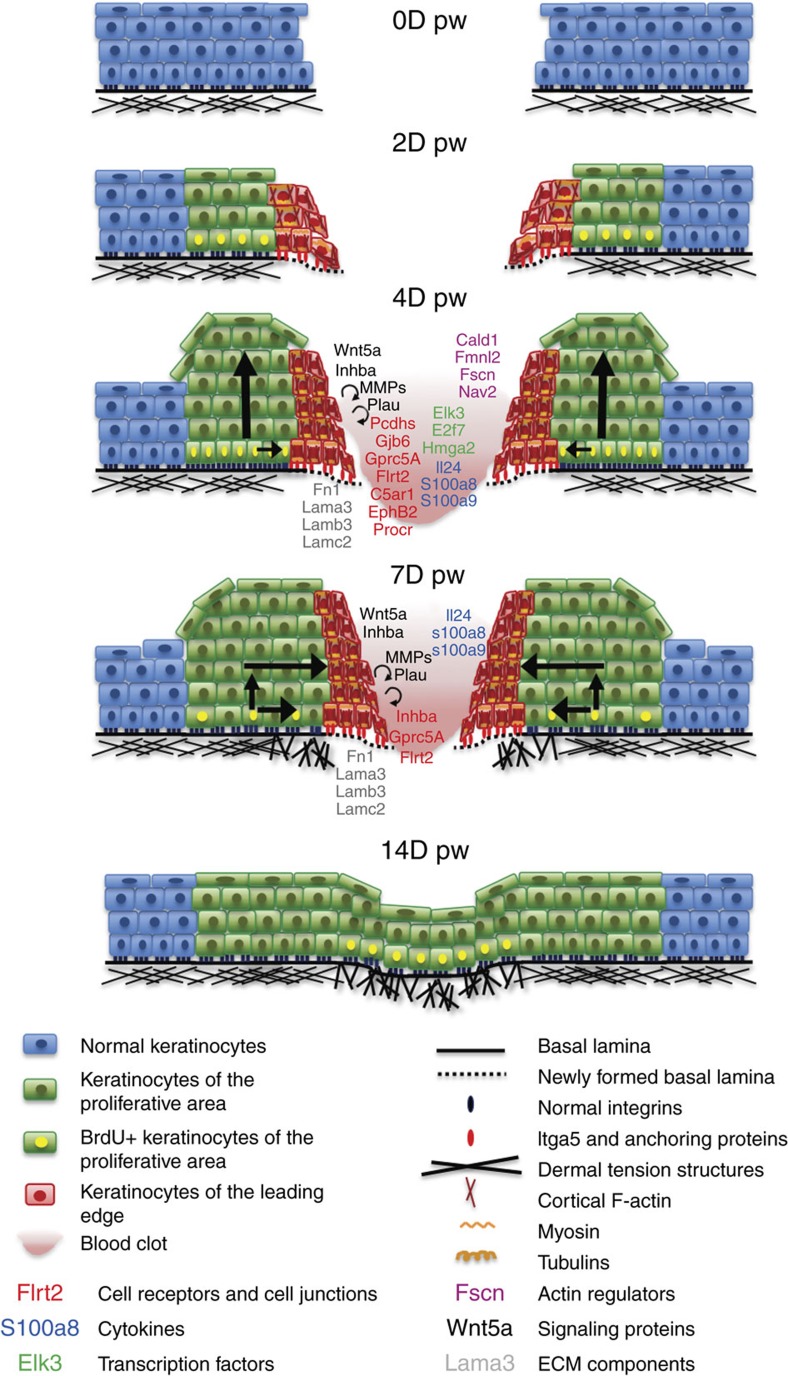
Model of WH in mice. Cartoons representing the different steps occurring during re-epithelialization of WH. Important genes expressed preferentially during wounding are highlighted. The arrows represent the movement of cells from the basal to the suprabasal compartment including their suprabasal migration.

Our study confirms the existence of two distinct epidermal zones during wound repair; a proliferative hub composed of the IFE and HF-derived SCs and their progeny and a LE composed of non-proliferative cells^3^, and uncovers the timing, gene expression signature and mechanisms that specify these two distinct compartments during WH (Fig. 9). We propose that the non-proliferative LE of the wound acts as a scaffold allowing a harmonious healing process, by creating a platform secreting high level of enzymes that remodel the surrounding ECM and fibrin clot allowing tissue regeneration to progress toward the centre of the wound and protecting the SCs and their progeny from the immediate vicinity of the wound front and infection.

A similar wound margin structure with elongated migrating cells has been described during the early stages of WH following incisional wound in humans^63^, supporting the notion that this mode of wound repair has been conserved during evolution. Further functional studies will be needed to refine the respective role of the genes identified here in the LE signature. The high level of expression of several of these genes in patients with chronic ulcers^64^,^65^ suggests that defects in the formation and/or function of this structure may induce wound-healing problems leading to chronic ulcer formation.

## Methods

### Mice

*K14CREER* transgenic mice were provided by Fuchs^66^. *Lrig1-CreERT2* mice were a kind gift from Jensen^67^. *Involucrin-CreERT2* were previously described^12^. *Rosaconfetti* mice were provided by Clevers^68^. *Rosa YFP*^69^ mice were obtained from Jackson Laboratory. All animals were mixed strains. No statistical methods were used to predetermine sample size. The experiments were not randomized. The investigators were not blinded to allocation during experiments and outcome assessment. Mice colonies were maintained in a certified animal facility in accordance with European guidelines. The experiments were approved by the local ethical committee (CEBEA).

### Targeting Confetti or YFP expression in wound experiments

For lineage tracing experiment, *K14CreER/RosaConfetti*, *Lrig1-CreERT2/RosaConfetti* and *Involucrin-CRERT2/Rosa-YFP* male and female adult (between 2 and 6 months old) mice were induced at 2 months with 0.03 mg g^−1^, 0.27 mg g^−1^ or 0.08 mg g^−1^ of TAM (Sigma-Aldrich), respectively, by intra-peritoneal (IP) injection. For K14-CreER and Lrig1-CreERT2 tracing, 2 weeks after TAM induction, mice were anesthetized (5% xylazine 10% ketamine in PBS) and circular pieces of epidermis were removed from the tail epidermis using a 3 mm diameter biopsy punch (Stiefel, Ireland). For *Involucrin-CRERT2/Rosa-YFP* mice the wound was performed 4 days after TAM injection to analyse the contribution of suprabasal cells. Each mouse was subjected to three different punches in the tail and at least five mice per time points were analysed.

### Proliferation experiments

For BrdU experiments, CD1 male and female adult (between 2 and 6 months old) mice were wounded, injected with one single injection IP of BrdU (50 mg kg^−1^ in PBS) at the different time points and killed 4 h after. For the quantification at least an area of 1.5 mm^2^ per region per wound was analysed with Imaris software (Bitplane) to determine the percentage of BrdU positive cells.

### Epidermal whole-mount and immunostaining

Pieces of skin tail surrounding the wound were incubated in PBS/EDTA (20 mM) on a rocking plate at 37 °C for 1 h. Epidermis was separated from the dermis using forceps as an intact sheet and washed two times with PBS. Pieces of epidermis were pre-fixed in 4% paraformaldehyde for 1 h at room temperature. Epidermis were rinsed two times with PBS for 5 min and conserved in PBS with 0.2% azide at 4 °C. For the immunofluorescence staining, the entire pieces of epidermis were incubated in blocking buffer (1% BSA, 5% horse serum, 0.8% Triton in PBS) for 3 h at room temperature on a rocking plate (100 r.p.m.). The samples were incubated in primary antibodies overnight at 4 °C. The primary antibodies used were the following: anti-Integrinβ4 (rat, 1:200, BD Biosciences), anti-K14 (chicken, 1:2,000, custom batch, Thermo Fischer), anti-GFP (rabbit, 1:200, Molecular Probes). Samples were then washed three times in PBS with 0.2% tween during 1 h and incubated in appropriate secondary antibodies diluted 1:400 in blocking buffer for 1 h at room temperature on the rocking plate. For BrdU staining, samples were incubated in HCl 1 M at 37 °C for 45 min, washed with PBS 0.2% tween, stained with anti-BrdU (rat, 1:200, Abcam) in blocking buffer and with appropriate secondary antibody. The following secondary antibodies were used: anti-rat, anti-chicken, anti-rabbit conjugated to AlexaFluor488 (Molecular Probes), to Rhodamine Red-X or to Cy5 (Jackson Immuno Research). Alexa488-conjugated phalloidin (Life Technologies) was used 1:200 in blocking buffer to visualize F-actin microfilaments. Nuclei were stained in Hoechst solution diluted 1:5,000 for 30 min and mounted in DAKO-mounting medium supplemented with 2.5% Dabco (Sigma). Immunostaining pictures of the whole mounts presented in the figures are representative images of at least five different experiments.

### Microscope image acquisition and measurements

All pictures of section immunostaining were acquired using the Axio Imager M1 Microscope, the AxioCamMR3 or MrC5 camera and using the Axiovision software (Carl Zeiss). Acquisitions were performed at room temperature using × 20 numerical aperture (NA) 0.4 (Carl Zeiss). All confocal images were acquired at room temperature with a LSM780 confocal system fitted on an AxioExaminer Z1 upright microscope equipped with C-Apochromat × 40/1.1 or Plan Apochromat × 25/0.8 water immersion objectives (Zeiss, Iena, Germany). Optical sections 512 × 512 pixels were collected sequentially for each fluorochrome. The data sets generated were merged and displayed with the ZEN2012 software (Zeiss). 3D reconstitution images were processed using Imaris (Bitplane) software.

### Histology and immunostaining on sections

Skin epidermis was removed from tailbone, embedded in OCT and kept at −80 °C. Sections of 6 μm were cut using a CM3050S Leica cryostat (Leica Mycrosystems). After fixation in 4% paraformaldehyde for 10 min at room temperature, tissues were washed three times in PBS for 5 min and incubated in blocking buffer (1% BSA, 5% Horse serum, 0, 2% Triton in PBS) for 1 h at room temperature. Primary antibodies were incubated overnight at 4 °C. Sections were rinsed three times in PBS and incubated with secondary antibodies and Hoechst in blocking buffer for 1 h at room temperature. Sections were again washed three times with PBS. The following primary antibodies were used: anti-K14 (chicken, 1:20,000, custom batch, Thermo Fischer); anti-K6 (rabbit, 1:6,000, Covance), anti-Flrt2/3 (rabbit, 1:100, Sigma); anti-Gprc5a (rabbit, 1:100, Sigma); anti-Myo1b (rabbit, 1:100, Sigma) and anti-α5-integrin (PE-conjugated rat, 1:200, BD or rabbit, 1:200, Abcam). For the anti-Tubb2 (rabbit, 1/1,000, Abcam) staining, fixation was performed in methanol at −20 °C for 2.5 min and the rest of the protocol was performed as described. The following secondary antibodies were used diluted to 1:400: anti-rabbit, anti-rat, anti-chicken conjugated to Alexa Fluor 488 (Molecular Probes), to rhodamine Red-X (Jackson Immunoresearch) or to Cy5 (Jackson Immunoresearch). Nuclei were stained in Hoechst solution (1:2,000) and slides were mounted in DAKO-mounting medium supplemented with 2.5% Dabco (Sigma). Immunostaining pictures of the skin sections presented in the figures are representative images of at least five different experiments.

### Dissociation of epidermal cells and cell sorting

The dermis and epidermis were removed from the tail bone and micro dissection was performed using two different sizes of punch biopsy: one punch of 4 mm in diameter was done, around the wound of 3 mm in diameter, to remove the LE zone and one punch of 6 mm in diameter was used to remove the proliferative centre, at D4 and D7 after wound. Another piece of skin was taken, as control, from a region far from the wounded area. The two replicate samples at each time points were a pull of five CD1 mice. The samples were incubated in HBSS (Gibco) 0,25% trypsin (Gibco) at 37 °C until the epidermis was separated from the dermis (30 min). Epidermis was then incubated on a rocking plate (100 r.p.m.) at room temperature for 5 min. Basal cells were mechanically separated from the epidermis by flushing 10 times under the epidermis. Tissues were then cut in small pieces with a scalpel and incubated again for 5 min on a rocking plate (100 r.p.m.) at room temperature. Trypsin was then neutralized by adding DMEM medium (Gibco) supplemented with 2% Chelex Fetal Calf Serum (FCS) and the cells were mechanically separated by pipetting 90 times and filtrated on 70 μm filter (Falcon). Cells were incubated in 2% FCS/PBS with primary antibodies for 30 min on ice, protected from the light, with shaking every 10 min. Primary antibodies were washed with 2% FCS/PBS and cells incubated for 30 min in APC-conjugated streptavidin (BD Biosciences), on ice, with shaking every 10 min. Living epidermal cells were gated by forward scatter, side scatter and negative staining for Hoechst dye. For the first microarray analysis, basal IFE and infundibulum cells were stained using PE-conjugated anti-α6-integrin (clone GoH3; 1/200, ebioscience) and bulge cells were stained with biotinylated CD34 (clone RAM34; 1:50, BD Biosciences). Basal cells from the IFE were targeted using CD34 negative and α6-integrin positive gating. For the second microarray analysis, FITC-conjugated anti-α6-integrin (CD49f) (clone GoH3: 1:200, ebioscience) and PE-conjugated anti-α5-integrin (CD49e) (clone 5H10-27, 1:200, BD Bioscience) and biotinylated CD34 antibodies were used. The cells were sorted using CD34 negative α6-integrin positive α5-integrin positive gating. Before sorting, the cells were filtered again on 70 μm filter (Falcon). Fluorescence-activated cell sorting analysis was performed using FACSAria I at high pressure (70 psi) and FACSDiva software (BD Biosciences).

### Microarray analysis

Sorted cells (300 cells per sample) were collected directly in 45 μl of lysis buffer (20 mM DTT, 10 mM Tris–HCl pH 7.4, 0.5% SDS, 0.5 μg μl^−1^ proteinase K). Samples were then lysed at 65 °C for 15 min and frozen. RNA isolation, amplification and microarray were performed in the Functional Genomics Core, Barcelona. cDNA synthesis, library preparation and amplification were performed as described^70^. Microarrays were then performed on Mouse Genome 430 PM strip Affymetrix array at IRB Functional Genomics Core (Barcelona, Spain). The data were normalized using RMA algorithm. The entire procedure was repeated in three technical independent samples. Genetic signatures were obtained by considering genes presenting a fold change greater or smaller than 2 or −2, respectively, in each replicates. The accession number for the microarray data are GEO: GSE76795 and GSE93638.

### 5-FU and dexamethasone experiments

For the 5-FU experiments, mice were treated shortly after wound surgery with Efudix 5% cream (Meda Pharma) applied topically on the upper part of the tail three times per day until the sacrifice. For the dexamethasone experiments, dexamethasone powder (Sigma) was resuspended at 1 mg ml^−1^ in ethanol 100% and diluted 5 × in sterile PBS. The mice were injected intraperitoneally once per day at the dose of 1 mg kg^−1^. The treatment started 2 days before the wound surgery and was sustained until the end of the experiment.

### Data availability

Data supporting the findings of this study are available within the article (and its Supplementary Information files) and from the corresponding author on reasonable request. The accession number for the microarray data are GEO: GSE76795 and GSE93638.

## Additional information

**How to cite this article:** Aragona, M. *et al*. Defining stem cell dynamics and migration during wound healing in mouse skin epidermis. *Nat. Commun.* **8,** 14684 doi: 10.1038/ncomms14684 (2017).

**Publisher's note:** Springer Nature remains neutral with regard to jurisdictional claims in published maps and institutional affiliations.

## Supplementary Material

Supplementary Information Supplementary Figures, Supplementary Note and Supplementary References

## References

[b1] Arwert E. N., Hoste E. & Watt F. M. Epithelial stem cells, wound healing and cancer. Nat. Rev. Cancer. 12, 170–180 (2012). 22362215 10.1038/nrc3217

[b2] Gurtner G. C., Werner S., Barrandon Y. & Longaker M. T. Wound repair and regeneration. Nature 453, 314–321 (2008). 18480812 10.1038/nature07039

[b3] Coulombe P. A. Wound epithelialization: accelerating the pace of discovery. J. Invest. Dermatol. 121, 219–230 (2003). 12880412 10.1046/j.1523-1747.2003.12387.x

[b4] Plikus M. V. . Epithelial stem cells and implications for wound repair. Semin. Cell Dev. Biol. 23, 946–953 (2012). 23085626 10.1016/j.semcdb.2012.10.001 PMC3518754

[b5] Tumbar T. . Defining the epithelial stem cell niche in skin. Science 303, 359–363 (2004). 14671312 10.1126/science.1092436 PMC2405920

[b6] Ito M. . Stem cells in the hair follicle bulge contribute to wound repair but not to homeostasis of the epidermis. Nat. Med. 11, 1351–1354 (2005). 16288281 10.1038/nm1328

[b7] Jaks V. . Lgr5 marks cycling, yet long-lived, hair follicle stem cells. Nat. Genet. 40, 1291–1299 (2008). 18849992 10.1038/ng.239

[b8] Jensen K. B. . Lrig1 expression defines a distinct multipotent stem cell population in mammalian epidermis. Cell Stem Cell 4, 427–439 (2009). 19427292 10.1016/j.stem.2009.04.014 PMC2698066

[b9] Levy V., Lindon C., Harfe B. D. & Morgan B. A. Distinct stem cell populations regenerate the follicle and interfollicular epidermis. Dev. Cell 9, 855–861 (2005). 16326396 10.1016/j.devcel.2005.11.003

[b10] Levy V., Lindon C., Zheng Y., Harfe B. D. & Morgan B. A. Epidermal stem cells arise from the hair follicle after wounding. Faseb J. 21, 1358–1366 (2007). 17255473 10.1096/fj.06-6926com

[b11] Snippert H. J. . Lgr6 marks stem cells in the hair follicle that generate all cell lineages of the skin. Science 327, 1385–1389 (2010). 20223988 10.1126/science.1184733

[b12] Mascre G. . Distinct contribution of stem and progenitor cells to epidermal maintenance. Nature 489, 257–262 (2012). 22940863 10.1038/nature11393

[b13] Chen L., Mirza R., Kwon Y., DiPietro L. A. & Koh T. J. The murine excisional wound model: contraction revisited. Wound Repair Regen. 23, 874–877 (2015). 26136050 10.1111/wrr.12338 PMC5094847

[b14] Ito M. . Wnt-dependent de novo hair follicle regeneration in adult mouse skin after wounding. Nature 447, 316–320 (2007). 17507982 10.1038/nature05766

[b15] Paladini R. D., Takahashi K., Bravo N. S. & Coulombe P. A. Onset of re-epithelialization after skin injury correlates with a reorganization of keratin filaments in wound edge keratinocytes: defining a potential role for keratin 16. J. Cell Biol. 132, 381–397 (1996). 8636216 10.1083/jcb.132.3.381 PMC2120730

[b16] Weiss R. A., Eichner R. & Sun T. T. Monoclonal antibody analysis of keratin expression in epidermal diseases: a 48- and 56-kdalton keratin as molecular markers for hyperproliferative keratinocytes. J. Cell Biol. 98, 1397–1406 (1984). 6201492 10.1083/jcb.98.4.1397 PMC2113245

[b17] Leigh I. M. . Keratins (K16 and K17) as markers of keratinocyte hyperproliferation in psoriasis *in vivo* and *in vitro*. Br. J. Dermatol. 133, 501–511 (1995). 7577575 10.1111/j.1365-2133.1995.tb02696.x

[b18] Wojcik S. M., Bundman D. S. & Roop D. R. Delayed wound healing in keratin 6a knockout mice. Mol. Cell Biol. 20, 5248–5255 (2000). 10866680 10.1128/mcb.20.14.5248-5255.2000 PMC85973

[b19] Schafer M. & Werner S. Transcriptional control of wound repair. Annu. Rev. Cell Dev. Biol. 23, 69–92 (2007). 17474876 10.1146/annurev.cellbio.23.090506.123609

[b20] Schelfhout V. R. . The role of heregulin-alpha as a motility factor and amphiregulin as a growth factor in wound healing. J. Pathol. 198, 523–533 (2002). 12434423 10.1002/path.1240

[b21] Poindexter N. J. . IL-24 is expressed during wound repair and inhibits TGFalpha-induced migration and proliferation of keratinocytes. Exp. Dermatol. 19, 714–722 (2010). 20545760 10.1111/j.1600-0625.2010.01077.x PMC3161412

[b22] Kerkhoff C. . Novel insights into the role of S100A8/A9 in skin biology. Exp. Dermatol. 21, 822–826 (2012). 22882537 10.1111/j.1600-0625.2012.01571.x PMC3498607

[b23] Barrandon Y. & Green H. Cell migration is essential for sustained growth of keratinocyte colonies: the roles of transforming growth factor-alpha and epidermal growth factor. Cell 50, 1131–1137 (1987). 3497724 10.1016/0092-8674(87)90179-6

[b24] Marchisio P. C., Bondanza S., Cremona O., Cancedda R. & De Luca M. Polarized expression of integrin receptors (alpha 6 beta 4, alpha 2 beta 1, alpha 3 beta 1, and alpha v beta 5) and their relationship with the cytoskeleton and basement membrane matrix in cultured human keratinocytes. J. Cell Biol. 112, 761–773 (1991). 1825212 10.1083/jcb.112.4.761 PMC2288862

[b25] Larjava H., Salo T., Haapasalmi K., Kramer R. H. & Heino J. Expression of integrins and basement membrane components by wound keratinocytes. J. Clin. Invest. 92, 1425–1435 (1993). 8376596 10.1172/JCI116719 PMC288287

[b26] Hertle M. D., Kubler M. D., Leigh I. M. & Watt F. M. Aberrant integrin expression during epidermal wound healing and in psoriatic epidermis. J. Clin. Invest. 89, 1892–1901 (1992). 1601996 10.1172/JCI115794 PMC295888

[b27] Guo M. . Altered processing of integrin receptors during keratinocyte activation. Exp. Cell Res. 195, 315–322 (1991). 2070815 10.1016/0014-4827(91)90379-9

[b28] Heller E., Kumar K. V., Grill S. W. & Fuchs E. Forces generated by cell intercalation tow epidermal sheets in mammalian tissue morphogenesis. Dev. Cell 28, 617–632 (2014). 24697897 10.1016/j.devcel.2014.02.011 PMC4041280

[b29] Stevens L. J. & Page-McCaw A. A secreted MMP is required for reepithelialization during wound healing. Mol. Biol. Cell 23, 1068–1079 (2012). 22262460 10.1091/mbc.E11-09-0745 PMC3302734

[b30] Hattori N. . MMP-13 plays a role in keratinocyte migration, angiogenesis, and contraction in mouse skin wound healing. Am. J. Pathol. 175, 533–546 (2009). 19590036 10.2353/ajpath.2009.081080 PMC2716954

[b31] Hartenstein B. . Epidermal development and wound healing in matrix metalloproteinase 13-deficient mice. J. Invest. Dermatol. 126, 486–496 (2006). 16374453 10.1038/sj.jid.5700084 PMC2767339

[b32] Madlener M., Parks W. C. & Werner S. Matrix metalloproteinases (MMPs) and their physiological inhibitors (TIMPs) are differentially expressed during excisional skin wound repair. Exp. Cell Res. 242, 201–210 (1998). 9665817 10.1006/excr.1998.4049

[b33] Okada A. . Expression of matrix metalloproteinases during rat skin wound healing: evidence that membrane type-1 matrix metalloproteinase is a stromal activator of pro-gelatinase A. J. Cell Biol. 137, 67–77 (1997). 9105037 10.1083/jcb.137.1.67 PMC2139851

[b34] Lund I. K. . Concomitant lack of MMP9 and uPA disturbs physiological tissue remodeling. Dev. Biol. 358, 56–67 (2011). 21802414 10.1016/j.ydbio.2011.07.021

[b35] Nunan R. . Ephrin-Bs drive junctional downregulation and actin stress fiber disassembly to enable wound re-epithelialization. Cell Rep. 13, 1380–1395 (2015). 26549443 10.1016/j.celrep.2015.09.085 PMC4660216

[b36] Hubner G., Hu Q., Smola H. & Werner S. Strong induction of activin expression after injury suggests an important role of activin in wound repair. Dev. Biol. 173, 490–498 (1996). 8606007 10.1006/dbio.1996.0042

[b37] Munz B. . Overexpression of activin A in the skin of transgenic mice reveals new activities of activin in epidermal morphogenesis, dermal fibrosis and wound repair. Embo J. 18, 5205–5215 (1999). 10508154 10.1093/emboj/18.19.5205 PMC1171591

[b38] Beer H. D. . Expression and function of keratinocyte growth factor and activin in skin morphogenesis and cutaneous wound repair. J. Investig. Dermatol. Symp. Proc. 5, 34–39 (2000). 10.1046/j.1087-0024.2000.00009.x 11147673

[b39] Wankell M. . Impaired wound healing in transgenic mice overexpressing the activin antagonist follistatin in the epidermis. Embo J. 20, 5361–5372 (2001). 11574468 10.1093/emboj/20.19.5361 PMC125651

[b40] Bamberger C. . Activin controls skin morphogenesis and wound repair predominantly via stromal cells and in a concentration-dependent manner via keratinocytes. Am. J. Pathol. 167, 733–747 (2005). 16127153 10.1016/S0002-9440(10)62047-0 PMC1698729

[b41] Antsiferova M. . Keratinocyte-derived follistatin regulates epidermal homeostasis and wound repair. Lab. Invest. 89, 131–141 (2009). 19079322 10.1038/labinvest.2008.120 PMC4087116

[b42] Lewis C. J. . Bone morphogenetic protein signaling suppresses wound-induced skin repair by inhibiting keratinocyte proliferation and migration. J. Invest. Dermatol. 134, 827–837 (2014). 24126843 10.1038/jid.2013.419 PMC3945401

[b43] Moll I., Houdek P., Schafer S., Nuber U. & Moll R. Diversity of desmosomal proteins in regenerating epidermis: immunohistochemical study using a human skin organ culture model. Arch. Dermatol. Res. 291, 437–446 (1999). 10482015 10.1007/s004030050435

[b44] Gallinaro H. . A 4.2kb upstream region of the human corneodesmosin gene directs site-specific expression in hair follicles and hyperkeratotic epidermis of transgenic mice. J. Invest. Dermatol. 122, 730–738 (2004). 15086560 10.1111/j.0022-202X.2004.22306.x

[b45] Tao Q. . Identification of the retinoic acid-inducible Gprc5a as a new lung tumor suppressor gene. J. Natl Cancer Inst. 99, 1668–1682 (2007). 18000218 10.1093/jnci/djm208

[b46] Chen Y. . Gprc5a deletion enhances the transformed phenotype in normal and malignant lung epithelial cells by eliciting persistent Stat3 signaling induced by autocrine leukemia inhibitory factor. Cancer Res. 70, 8917–8926 (2010). 20959490 10.1158/0008-5472.CAN-10-0518 PMC2970717

[b47] Zhong S. . Lung tumor suppressor GPRC5A binds EGFR and restrains its effector signaling. Cancer Res. 75, 1801–1814 (2015). 25744720 10.1158/0008-5472.CAN-14-2005

[b48] Endo-Munoz L. . E2F7 can regulate proliferation, differentiation, and apoptotic responses in human keratinocytes: implications for cutaneous squamous cell carcinoma formation. Cancer Res. 69, 1800–1808 (2009). 19223542 10.1158/0008-5472.CAN-08-2725

[b49] Blanpain C., Lowry W. E., Geoghegan A., Polak L. & Fuchs E. Self-renewal, multipotency, and the existence of two cell populations within an epithelial stem cell niche. Cell 118, 635–648 (2004). 15339667 10.1016/j.cell.2004.08.012

[b50] Yamagishi S. . FLRT2 and FLRT3 act as repulsive guidance cues for Unc5-positive neurons. Embo J. 30, 2920–2933 (2011). 21673655 10.1038/emboj.2011.189 PMC3160250

[b51] Sun B. K., Siprashvili Z. & Khavari P. A. Advances in skin grafting and treatment of cutaneous wounds. Science, 346, 941–945 (2014). 25414301 10.1126/science.1253836

[b52] Sanchis A. . Keratinocyte-targeted overexpression of the glucocorticoid receptor delays cutaneous wound healing. PLoS ONE 7, 1–10 (2012). 10.1371/journal.pone.0029701 PMC3250471 22235328

[b53] Blanpain C. & Simons B. D. Unravelling stem cell dynamics by lineage tracing. Nat. Rev. Mol. Cell Biol. 14, 489–502 (2013). 23860235 10.1038/nrm3625

[b54] Sánchez-Danés A. . Defining the clonal dynamics leading to mouse skin tumour initiation. Nature 536, 298–303 (2016). 27459053 10.1038/nature19069 PMC5068560

[b55] Doupe D. P. . A single progenitor population switches behavior to maintain and repair esophageal epithelium. Science 337, 1091–1093 (2012). 22821983 10.1126/science.1218835 PMC3527005

[b56] Roshan A. . Human keratinocytes have two interconvertible modes of proliferation. Nat. Cell Biol. 18, 145–156 (2016). 26641719 10.1038/ncb3282 PMC4872834

[b57] Fu X., Sun X., Li X. & Sheng Z. Dedifferentiation of epidermal cells to stem cells *in vivo*. Lancet 358, 1067–1068 (2001). 11589942 10.1016/S0140-6736(01)06202-X

[b58] Mannik J., Alzayady K. & Ghazizadeh S. Regeneration of multilineage skin epithelia by differentiated keratinocytes. J. Invest. Dermatol. 130, 388–397 (2010). 19675579 10.1038/jid.2009.244 PMC2879264

[b59] Lescroart F. . Early lineage restriction in temporally distinct populations of Mesp1 progenitors during mammalian heart development. Nat. Cell Biol. 16, 829–840 (2014). 25150979 10.1038/ncb3024 PMC6984965

[b60] Chabab S. . Uncovering the number and clonal dynamics of Mesp1 progenitors during heart morphogenesis. Cell Rep. 14, 1–10 (2016). 26725109 10.1016/j.celrep.2015.12.013 PMC4709258

[b61] Wuidart A. . Quantitative lineage tracing strategies to resolve multipotency in tissue-specific stem cells. Genes Dev. 30, 1261–1277 (2016). 27284162 10.1101/gad.280057.116 PMC4911926

[b62] Clayton E. . A single type of progenitor cell maintains normal epidermis. Nature 446, 185–189 (2007). 17330052 10.1038/nature05574

[b63] Odland G. & Ross R. Human wound repair. I. Epidermal regeneration. J. Cell Biol. 39, 35–151 (1968). 5678445 10.1083/jcb.39.1.135 PMC2107509

[b64] Rayment E. A., Upton Z. & Shooter G. K. Increased matrix metalloproteinase-9 (MMP-9) activity observed in chronic wound fluid is related to the clinical severity of the ulcer. Br. J. Dermatol. 158, 951–961 (2008). 18284390 10.1111/j.1365-2133.2008.08462.x

[b65] Shih B. . Identification of biomarkers in sequential biopsies of patients with chronic wounds receiving simultaneous acute wounds: a genetic, histological, and noninvasive imaging study. Wound Repair Regen. 20, 757–769 (2012). 22985042 10.1111/j.1524-475X.2012.00832.x

[b66] Vasioukhin V., Degenstein L., Wise B. & Fuchs E. The magical touch: genome targeting in epidermal stem cells induced by tamoxifen application to mouse skin. Proc. Natl Acad. Sci. USA 96, 8551–8556 (1999). 10411913 10.1073/pnas.96.15.8551 PMC17554

[b67] Page M. E., Lombard P., Ng F., Gottgens B. & Jensen K. B. The epidermis comprises autonomous compartments maintained by distinct stem cell populations. Cell Stem Cell 13, 471–482 (2013). 23954751 10.1016/j.stem.2013.07.010 PMC3793873

[b68] Snippert H. J. . Intestinal crypt homeostasis results from neutral competition between symmetrically dividing Lgr5 stem cells. Cell 143, 134–144 (2010). 20887898 10.1016/j.cell.2010.09.016

[b69] Srinivas S. . Cre reporter strains produced by targeted insertion of EYFP and ECFP into the ROSA26 locus. BMC Dev. Biol. 1, 4 (2001). 11299042 10.1186/1471-213X-1-4 PMC31338

[b70] Gonzalez-Roca E. . Accurate expression profiling of very small cell populations. PLoS ONE 5, e14418 (2010). 21203435 10.1371/journal.pone.0014418 PMC3010985

