# Supplementary material for: Defining stem cell dynamics and migration during wound healing in mouse skin epidermis
Source: Nat Commun. 2017 Mar 1;8:14684. doi: 10.1038/ncomms14684 (PMC5339881; doi:10.1038/ncomms14684)
Supplement: Supplementary Information — Supplementary Figures, Supplementary Note and Supplementary References [file ncomms14684-s1.pdf]

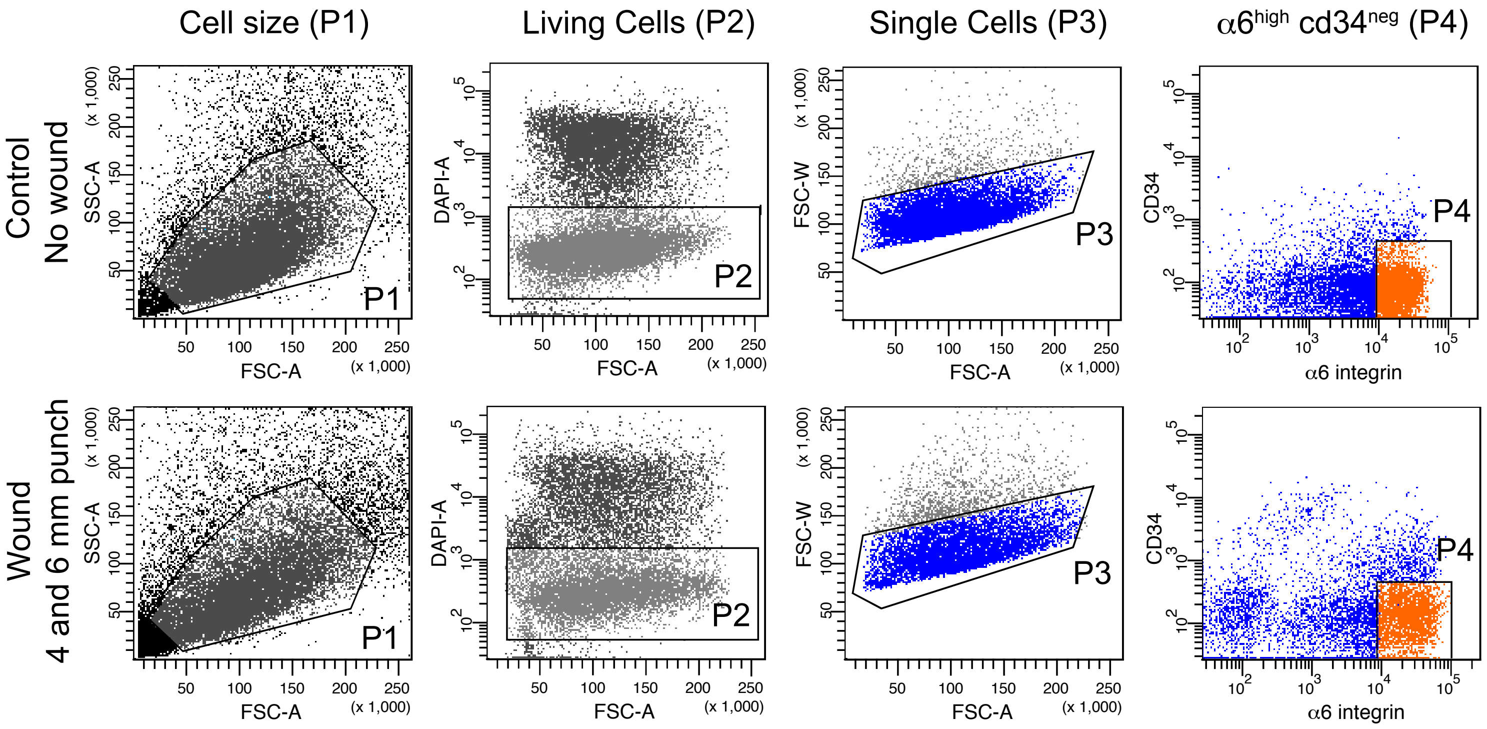

Supplementary Figure 1. FACS strategy used to isolate basal cells from the LE and the proliferative hub 4 and 7 days after wound.

Representative FACS plots showing the strategy used to isolate basal cells from the LE and the Proliferative hub. 4 and 7 days after wound, pieces of tail skin located far from the wound (control) and surrounding the wound obtained with the 6mm (proliferative hub) and the 4mm (leading edge) punches were dissociated by enzymatic and mechanical separation. Isolated epidermal cells were stained with α 6 integrin and CD34 antibodies. Single living cells were gated by debris exclusion (P1), DAPI exclusion (P2), doublet elimination (P3) and basal IFE α6^{high} CD34^{neg} cells were sorted (P4).

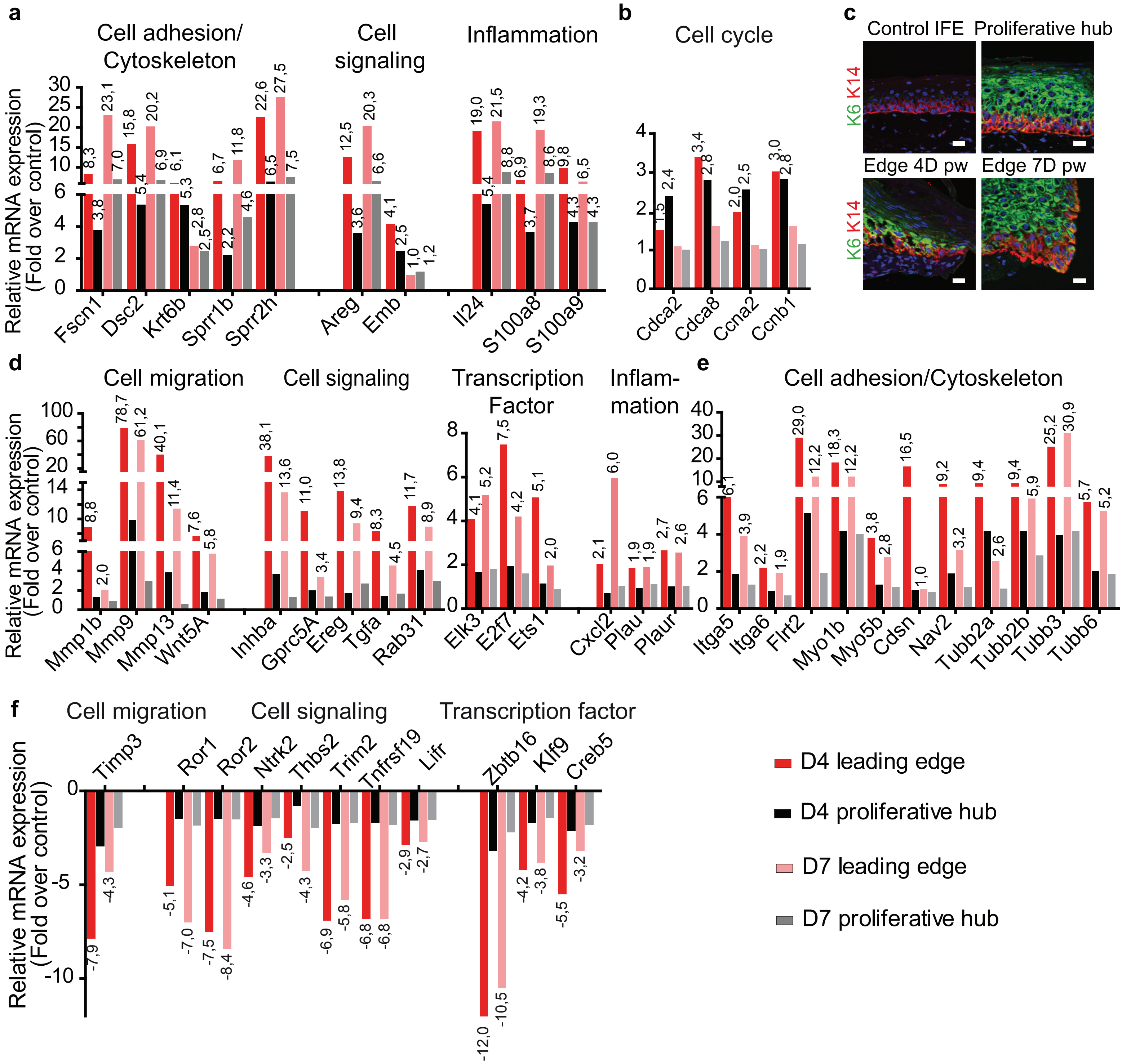

Supplementary Figure 2. Gene expression in the proliferative hub and leading edge as a wound signature.

a-**b**, Relative expression measured by microarray of genes upregulated in both proliferative hub and LE 4 and 7 days after wound. These genes are implicated in cell adhesion and cytoskeleton, cell signaling, inflammation (**a**) or cell cycle regulation (**b**). The fold changes are presented over the control (n=2). **c**, Immunofluorescence showing the expression of K6 (green) and K14 (red) on sections of epidermis in the proliferative hub and the leading edge 4 and 7 days after wound. Scale bar = 20µm. **d**-**e**, Relative expression measured by microarray of genes more specifically up regulated in the leading edge compared to the proliferative hub 4 and 7 days after wound. The genes are implicated in cell migration, cell signaling, transcription, inflammation (**d**) as well as cell adhesion and cytoskeleton (**e**). The fold changes are presented over the control (n=2). **f**, Relative expression of genes down regulated in the leading edge compared to the proliferative hub 4 and 7 days after wound. The fold changes are presented over the control (n=2).

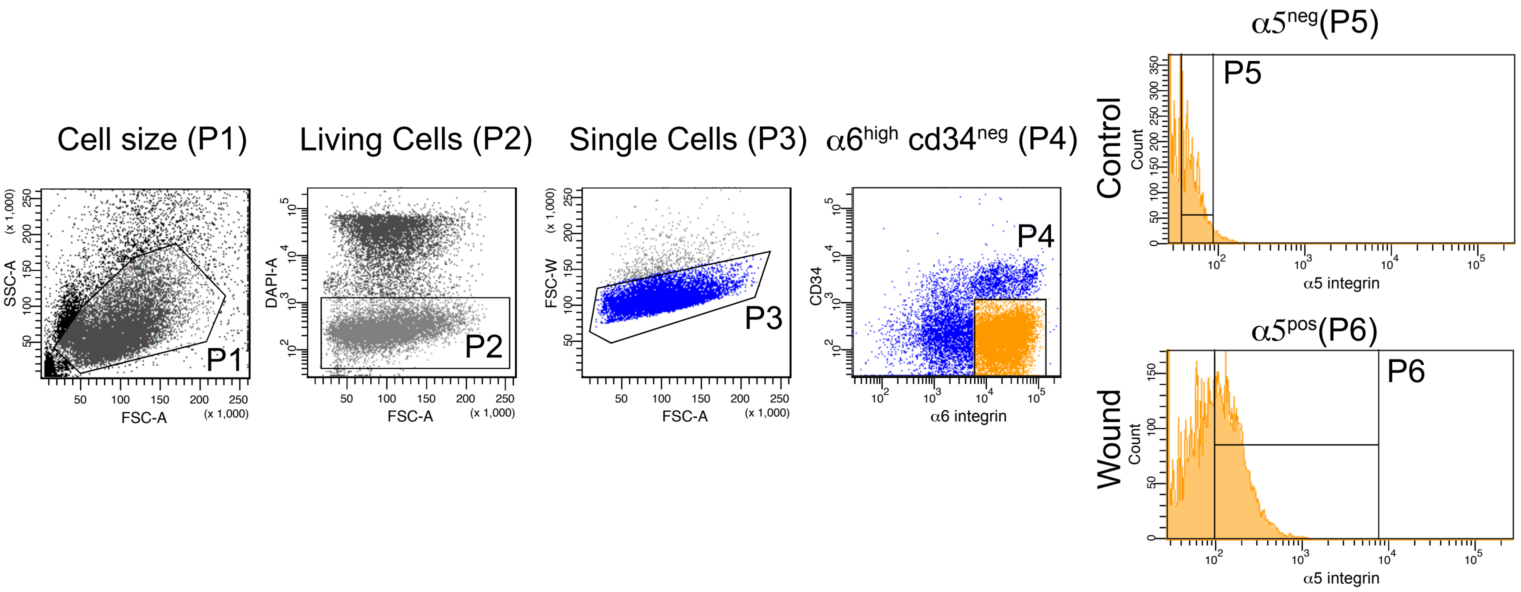

Supplementary Figure 3. FACS strategy used to isolate LE basal cells using their α5 integrin specific expression 4 days after wound.

Representative FACS plots showing the strategy used to isolate LE basals cells based on α5 integrin expression. 4 days after wound, pieces of tail skin located far from the wound (control) and surrounding the wound obtained with the 4mm punch were dissociated by enzymatic and mechanical separation. Isolated epidermal cells were stained with α5 integrin, α6 integrin and CD34 antibodies. Single living cells were gated by debris exclusion (P1), DAPI exclusion (P2), doublet elimination (P3). Within the $α6^{high}$ $CD34^{neg}$ gate (P4), basal IFE cells were sorted according to their α5 integrin expression.

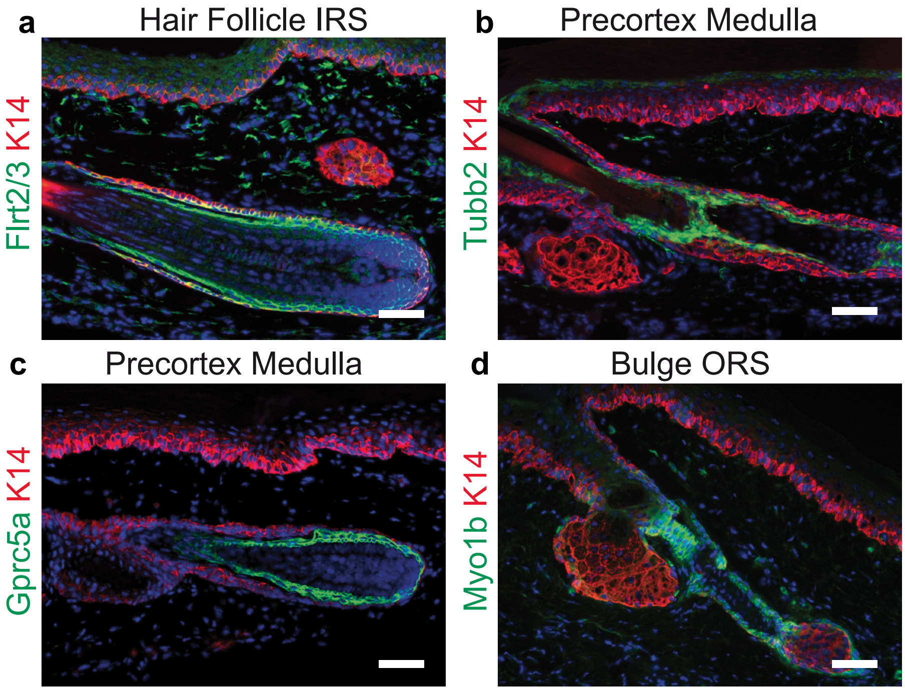

Supplementary Figure 4. Expression of the LE markers in the hair follicle.

a-**d**, Immunofluorescence of the wound markers (green) in normal unwounded skin. Flrt2/3 is expressed in the inner root sheath (IRS) of the normal hair follicle (**a**), Tubb2 and Gprc5a are expressed in the precortex medulla (**b**-**c**) and Myo1b is expressed in the bulge outer root sheet (ORS) (**d**). In all the pictures K14 is in red and the nuclei are stained with Hoechst (blue). Scale bar =50μm.

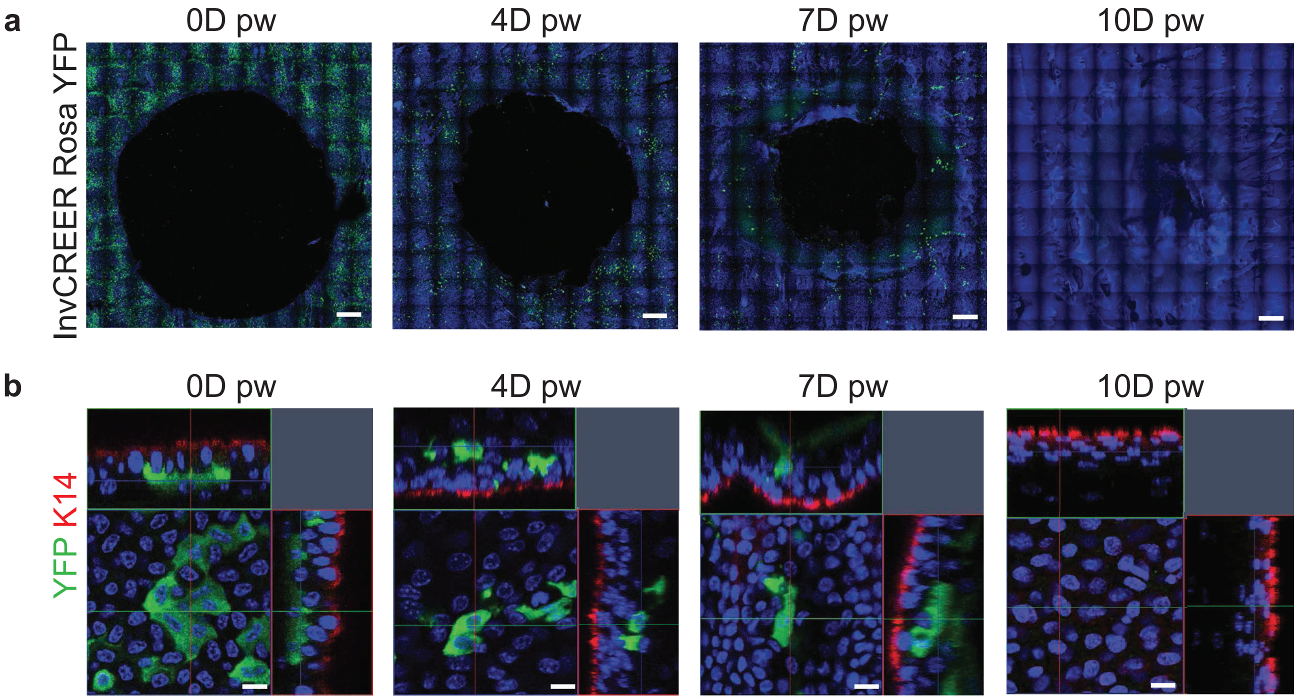

Supplementary Figure 5. Suprabasal cells do not revert back to basal cells during WH.

a, Maximum intensity projection of representative confocal pictures showing YFP immunostaining performed on whole-mount of wounded tail epidermis 4 days after TAM administration to *InvCREER/RosaYFP* mice and analysed at different times following wounding. Most of the Inv suprabasal cells are lost during the healing process. Scale bar = 500µm. **b**, Representative confocal pictures of YFP (green) and K14 (red) expression in *InvCREER/RosaYFP* mice in whole mount immunostaining at the different time points following wounding, showing the departure of the suprabasal Inv YFP labelled clones from the IFE. Scale bar = 20µm.

Supplementary Note 1

In this Supplementary Note we present further details of the biophysical and statistical approach used to infer the lineage hierarchy and proliferation kinetics of progenitors involved in skin wound healing. By taking into account the potential frequency of mergers we estimate the fraction of K14CREER cells contributing to wound healing. We then make use of stochastic modelling approach to infer the simplest model which is capable of describing the broad range of clonal dynamics observed in experiment.

Fraction of K14CREER progenitors contributing to wound healing

We begin our analysis by determining the fraction of K14CREER cells contributing to skin regeneration. To this end we first note that if labelling of K14CREER cells is statistically independent and representative this fraction is equal to the fraction of *labelled* K14CREER cells contributing to the wound. Denoting by n the number of labelled clones involved in wound healing and by N the total number of labelled clones that are capable of wound healing this fraction is given by $f = n/(mN)$. Here, m is the average number of basal cells in K14CREER clones. For the purpose of this analysis we define clones that contribute to wound healing as unicolour stripe-like structures that radially extend from the tissue surrounding the wound towards the centre of the wound. As the wound in most samples is closed at day 14 post wounding the number of stripes in a given wound is a proxy for n. The analysis of the experimental data is complicated by the relatively high induction frequency and the possibility that a given stripe is the merger of two different clones. To correct for possible merging events we therefore calculate the probability of merging for stripes of

a given colour c. If induction and stripe formation are each statistically independent and uniformly distributed around the wound the probability that neighbouring stripes are located at a distance d is $g(d) = \rho_c \exp(-\rho_c d)$, where ρ_c is the density of stripes of colour c . Then, if w is the typical width of stripes, the probability that nearest neighbours overlap is $G^c_{\text{unicolour}} = 1 - \exp(\rho_c w)$. If the density of stripes is low, $\rho_c \ll 1$, we can approximate the merging probability by expanding the exponential,

$$G^c_{\text{unicolour}} \approx \rho_c w \, . \tag{1}$$

As we do not know ρ_c we proceed by investigating merging events of stripes in different colours. The probability that clones of different colours merge is equal to the probability of mergers between any two colours times the probability that two stripes are of different colour,

$$G_{\text{bicolour}} \approx \rho_{\text{tot}} w \left(1 - \sum_c r_c^2 \right) \tag{2}$$

Here, r_c is the relative frequency of colour c, which we can calculate from relative colour abundances in the control region and $\rho_{\text{tot}} = \sum_c \rho_c$. We can solve this equation for w and substitute into the expression for $G^c_{\text{unicolour}}$ to obtain

$$G^c_{\text{unicolour}} \approx \frac{G_{\text{bicolour}}}{1 - \sum_c r_c^2} \frac{\rho_c}{\rho_{\text{tot}}} \, . \tag{3}$$

The second fraction on the right hand side is the ratio of the density of stripes labelled in colour c and the total density of labelled stripes. This ratio is equal to the relative recombination frequency of colour $\rho_c/\rho_{\text{tot}} = r_c$, and it can therefore be calculated from the control region.

To infer the frequency of mergers of a given colour we still need to calculate the fraction of bicolour mergers, G_{bicolour}, from the experimental data. This fraction is simply the number of bicolour

mergers of any colour combination, n_{bicolour}, divided by to total number of stripes. For the latter we again need to correct for the possibility of mergers and obtain

$$G_{\text{bicolour}} \approx \frac{n_{\text{bicolour}}}{n_{\text{stripes}}\left(1+\sum_c r_c G^c_{\text{unicolour}}\right)}\,. \tag{4}$$

Substituting this into the expression for $G^c_{\text{unicolour}}$ we obtain

$$G^c_{\text{unicolour}} + G^c_{\text{unicolour}} \sum_c r_c G^c_{\text{unicolour}} \approx \frac{n_{\text{bicolour}} r_c}{n_{\text{stripes}}\left(1-\sum_c r_c^2\right)}\,. \tag{5}$$

To proceed we again note that ρ_c is small, such that we may restrict our analysis to the highest order contributions in ρ_c. As $G^c_{\text{unicolour}}$ is proportional to ρ_c the first term on the left hand side is of order ρ_c while the other terms on the left hand side are of order ρ_c^2 and can therefore be neglected. By not taking into account these terms we slightly overestimate the frequency of mergers. We finally obtain the following approximate expression for the frequency of mergers in colour c,

$$G^c_{\text{unicolour}} \approx \frac{n_{\text{bicolour}} r_c}{n_{\text{stripes}}\left(1-\sum_c r_c^2\right)}\,. \tag{6}$$

Calculating the frequency of mergers for the different colours in all wounds we found that mergers are relatively rare, constituting on average less than 10% of all unicolour stripes (Extended Data Fig. 2d). With this, the total number of stripes in a given colour is $n^c = n^c_{\text{stripes}}\left(1+\sum_{c'} r^{c'} G^{c'}_{\text{unicolour}}\right)$. Summation over all colours finally gives $n = n_{\text{stripes}}\left(1+\sum_c r^c G^c_{\text{unicolour}}\right)$.

We now calculate the number of labelled K14CREER cells in the recruitment area around the wound. To this end we multiply the density of clones at the time of initiation of stripes, ρ_0, with the recruitment area, A, from which K14CREER cells can be activated to form stripes: $N = \rho_0 A$. We quantified the clonal density in the recruitment area at day 4 post wounding, when stripe formation

is initiated. We found that, on average, roughly 306 ± 84 (95% confidence intervals) K14CREER clones populate an area of 1mm^2. We defined the recruitment area by the ring spanned by the 10th and the 90th percentiles of the distances between the starting positions of stripes to the center of the wound. As we took these positions at day 14 post wounding we corrected these distances for the contraction of the tissue surrounding the wound. We found that until day 14 tissue contraction led to a 30% decrease of the distance between the hair follicles and the centre of the wound (Figure 1f of the main text). We therefore increased these distances by 30% to take account for the fact that the recruitment area was larger at the time of initiation than at day 14. With this we found that the recruitment area is roughly $16\ \text{mm}^2$, corresponding to a ring of width 1 mm around the boundary of the wound. Therefore, on average 4853 ± 1336 clones were labelled at day 4 in the recruitment area. With an average number of basal cells in these clones of 0.67 ± 0.16 we find that, on average, 3242 ± 868 basal cells were labelled at the initiation of stripe formation.

Taken together we obtain that $0.2 \pm 0.2\%$ of K14CREER cells contribute to wound healing by forming stripes. To calculate confidence intervals we employed standard methods for the propagation of uncertainty. It is important to note that the confidence intervals we report here do not cover the full uncertainty, as they necessarily do not take into account sources of uncertainty that are difficult to quantified. For example the uncertainty associated with the definition of the time point of stripe initiation (here taken as day 4 post wounding) depends on the unknown clonal dynamics in this time interval.

Number of stripes contributing to wound healing

Having determined the fraction of K14CREER basal cells that contribute to wound healing we now investigate the total number of these cells. To begin, we first ask how many progenitors are hypothetically needed to fill the wound. At day 4 post wounding the radius of the wound is roughly 1 mm. At day 14 post wounding we measured, on average, 12089 ± 1120 basal cells per mm^2, which means that roughly 38000 basal cells are needed for wound healing. To the contribution of a single stripe to wound healing we then counted the number of basal and suprabasal cells in stripes. While the frequency of mergers is relatively low these events can have a large impact on the average number of cells in clones. To take into account the possibility of mergers we identified, based on their morphology and location, stripes which we were confident to be monoclonal. The average number of basal cells in the remaining monoclonal stripes at day 14 is 19 ± 7, suggesting that if wound healing was entirely achieved by stripe formation 2054 ± 754 stripes were needed.

We compare this number to the total number of stripes that contribute to wound healing. Based on the number of labelled stripes, n, the total number of stripes is n/p_{ind}, where p_{ind} is the probability that a K14CREER cell is induced at day 4. The latter can be obtained by dividing the density of labelled clones in the recruitment area at day 4 by the density of basal cells, which we obtained from an uninjured control region. With this, in stark contrast to the number of stripes needed to regenerate the tissue, we found that in total 343 ± 120 stripes contribute to wound healing. This discrepancy suggests that other subpopulations contribute to wound healing, for example Lrig1CREER cells located at the hair follicles. It is important to note, however, that the labelling

is likely not representative and we don't know the relative induction of basal stem and progenitor cells. If the proportion of labelled progenitors differs significantly from that found in previous studies [1] this number might be significantly higher.

Stochastic modelling of clonal dynamics

To define cell fate behaviour in skin wound healing we extend the biophysical modelling approach introduced in Refs. [2–4]. In this model, stem cells and their differentiating progenitor cell progeny function as equipotent cell populations in which their proliferation kinetics and fate behaviour follow defined statistical dependencies. These stochastic "rules" summarize the product of the complex gene regulatory processes that regulate cell fate behaviours. For simplicity, following Refs. [2,3], we consider a model based on intrinsic (cell-autonomous) regulation. However, in the two-dimensional geometry of the epidermis, models of fate behaviour based on external (cell extrinsic) regulation are difficult to discriminate from intrinsically regulated systems. The clonal analysis described here therefore leaves the question of the underlying regulatory dynamics unspecified.

Maximum likelihood estimation

In infer cell fate behaviour in skin wound healing we employ *Bayesian inference* [5]. In particular, we seek to define the model which describes the experimental data with the highest probability. A detailed description of this approach is given in Ref. [4]. Briefly, the probability $P(\Theta|E)$ of a model, Θ given the experimental evidence E is, following *Bayes' Theorem*, proportional to the product of

the probability of obtaining the experimental evidence given the validity of the model, $P(E|\Theta)$, and the *a priori* belief in a certain model, $P(\Theta)$:

$$P(\Theta|E) = \frac{P(E|\Theta)P(\Theta)}{\int_{\Theta} \mathrm{d}\,\Theta P(E|\Theta)P(\Theta)}\,. \tag{7}$$

If the prior distribution $P(\Theta)$ is uniform we can infer the best fitting model by maximising the likelihood function $\mathcal{L}_E(\Theta) \equiv P(E|\Theta)$.

With the frequency $f_{n,t}$ of observations of clones with given numbers of basal cells n at a given time point t after wounding and since measurements are statistically independent, the likelihood function takes the form of a multinomial distribution [4],

$$\mathcal{L}_E(\Theta) = \frac{\left(\sum_{n,t} f_{n,t}\right)!}{\prod_{n,t} f_{n,t}!} \prod_t \prod_n P(n,t|\Theta)^{f_{n,t}}. \tag{8}$$

To obtain approximations for $P(n,t|\Theta)$ we numerically solved *Master equations* of the form

$$\frac{\mathrm{d}}{\mathrm{d}t} P(n,t|\Theta) = \sum_{n'=0}^{\infty} \mathcal{W}_{n',n} P(n',t|\Theta) - \mathcal{W}_{n,n'} P(n,t|\Theta), \tag{9}$$

using *Gillespie's algorithm* [6]. These solutions of the Master equations give the probability to find any stochastic fate of a single clone. From this we obtain the distribution of persisting clones as

$$P^p_{n>0}(n,t|\Theta) = \frac{P(n,t|\Theta)}{1 - P(0,t|\Theta)}\,. \tag{10}$$

With this, the logarithm of the likelihood function is given by

$$\log \mathcal{L}_E(\Theta) = \log \mathcal{N}^{-1} + \sum_t \sum_n N_{n,t} \log P^p_{n>0}(n,t|\Theta)\,. \tag{11}$$

To determine the maximum of the likelihood function we rasterised parameter space determine the best fit parameters and to calculate credible intervals, as defined below.

To estimate the uncertainty associated with the maximum likelihood parameters define credibility intervals comprising all parameter value whose likelihood is above a threshold, $\alpha = 0.05$, such that the resulting credible region (CR) is

$$\text{CR} = \left\{ \Theta \;\middle|\; \frac{\mathcal{L}_E(\Theta)}{\mathcal{L}_E(\Theta^*)} > \alpha \right\} . \tag{12}$$

This approach is most powerful in classifying sets of credible parameters [5]. For a specific model parameter, Θ_i, we report credible intervals in the form $\Theta_i^* \pm (\max_{\text{CR}}\{\Theta_i\} - \Theta_i^*, \Theta_i^* - \min_{\text{CR}}\{\Theta_i\})$.

Inference of the best fitting model

We are now in a position to perform maximum likelihood estimation in order to estimate the model, which describes the experimental data with the highest likelihood. The clonal analysis is complicated by the fact that skin wound healing comprises two temporal regimes: an early, highly proliferative phase with a strong bias towards the suprabasal layer is followed by a phase where overall proliferation reaches lower levels and where stripe formation is observed. While we here study the second regime, the clonal data might be compromised by labelled remainders from the early dynamics. Indicative of this is the large fraction of clones containing only suprabasal cells at day 4 and 7 post wounding as well as the large clonal loss rate. To avoid the presence of these clones distorting the results we focussed on clones containing basal cells and fitted the model to the basal compartment only.

To unveil the clonal dynamics constituting stripe formation we seek to define the simplest model which is capable of predicting the clonal data. To begin we study the time evolution of the average number of basal cells in K14CREER clones. We find that this increase is surprisingly linear. At first sight this could indicate neutral dynamics of a single progenitor population, where loss of clones due to symmetric differentiation is balanced by the growth of surviving clones [2,3]. A model comprising neutral dynamics has been proposed for the clonal dynamics in homeostatic epidermis [7]. However, in this modelling scheme the rate of increase of the average number of basal cells is given by the rate of symmetric proliferative divisions, which would correspond corresponding to an unreasonable cell cycle time of less than six hours. Further, neutral dynamics would give rise to an equally high rate of loss of stripes. In the clonal data there is no clear trend indicating a decreasing number of stripes over time (3.7 ± 1.6, 14.0 ± 7.6 , 6.7 ± 2.3 for days 7, 10, and 14pw, respectively).

To understand the rapid linear increase in the average number of basal cells we considered a situation where the neutral dynamics in the intact skin arises from an asymmetrically dividing subpopulation in the basal layer. Such a model can be summarised as

$$A \xrightarrow{\alpha} AB\,, \tag{13}$$

$$B \xrightarrow{\beta_1} BB\,, \tag{14}$$

$$B \xrightarrow{\beta_2} BC\,, \tag{15}$$

$$B \xrightarrow{\beta_3} CC\,, \tag{16}$$

where A is an asymmetrically dividing basal stem cell and B is a basal progenitor. C denotes

suprabasal cells. As the third process does not change the number of basal cells in a clone we cannot infer the rate β_2 from the basal data alone.

The time evolution of the probability to find a clone with n_B basal cells is described by Master equations of the form [8]

$$\frac{\mathrm{d}}{\mathrm{d}t}P(n_B,t) = \left[\alpha+\beta_1(n_B-1)\right]P(n_B-1,t)+\beta_3(n_B+1)P(n_B+1,t) \tag{17}$$

$$-\left[\alpha+(\beta_1+\beta_3)n_B\right]P(n_B,t)\,. \tag{18}$$

The solution has been shown to be a negative binomial distribution,

$$P(n_B,t) = \binom{\alpha+n_B-1}{n_B}(1-b_t)^{\alpha}b_t^{n_B}\,, \tag{19}$$

with the time dependent parameter

$$b_t = \frac{\exp[(\beta_1-\beta_3)t]-\beta_1}{\exp[(\beta_1-\beta_3)t]-\beta_3}\,. \tag{20}$$

The average number of basal cells follows

$$\langle n_B(t)\rangle = \frac{\alpha}{\beta_1-\beta_3}\left[e^{(\beta_1-\beta_3)t}-1\right]\,. \tag{21}$$

Therefore, if differentiation of basal progenitors is balanced with proliferation, $\beta_1=\beta_3\equiv\beta$, the average number of basal cells increases linearly with time,

$$\langle n_B(t)\rangle = \alpha t\,, \tag{22}$$

and the rate of increase is given by the division rate of basal stem cells.

A model comprising an asymmetrically dividing subpopulation which gives rise to balanced basal progenitors is therefore capable of predicting the time evolution of the average number of basal

cells in clones. The more challenging test is whether such a model is also able to predict the full distributions. We employed maximum likelihood estimation to infer the parameters that have the highest probability in describing the experimental data. We found that according to the so defined model basal progenitors divide asymmetrically with a rate $\alpha = 1.8 \pm (0.6, 0.4)\, d^{-1}$. Therefore, basal stem cells divide, on average, once every 13 hours. The most likely rates of symmetric proliferative and differentiating divisions of basal progenitors are $\beta = 1.3 \pm (1.1, 0.6)\, d^{-1}$, while the rate of asymmetric divisions of these cells cannot be inferred. Given that maximum likelihood estimation predicts a cell cycle time of at least 17 hours by symmetric divisions alone it is unlikely that, as in healthy epidermis, the majority of divisions is asymmetric. Notably, such a model resembles the clonal dynamics in the intact epidermis, where the basal compartment is comprised of basal stem cells, who divide mostly asymmetrically, and committed progenitors, who have a higher probability of symmetric divisions [1,9].

Having determined the clonal dynamics of K14CREER basal cells we then asked whether Lrig1CREER cells follow similar patterns of fate choice. The average number of basal cells in Lrig1CREER clones is significantly larger than in K14CREER clones. Remarkably, in common with the K14CREER clones this average increases linearly in time, suggesting that Lrig1CREER cells might show qualitatively similar behaviour. In principle, the higher rate of increase in the basal clone size might simply be the result of a reduced cell cycle time in Lrig1CREER cells. However, we found that in such a scenario the experimental data could again only be explained by assuming unreasonably fast division rates. Rather, we hypothesised that the increased size of Lrig1CREER clones might be the result of an initial round of symmetric divisions of basal stem cells. We approximate the

number of basal stem cells after the initial set of symmetric divisions by a Poisson distribution, such that the clone size distribution follows

$$\tilde{P}(n_B, t) = \sum_{k=1}^{\infty} \frac{\lambda^k}{k!} e^{-\lambda} P(n_B, t)^{*k}, \tag{23}$$

where the exponent denotes a k-fold convolution:

$$P(n_B, t)^{*k} = \sum_{n_B^1=1}^{n_B} P(n_B^1, t) \sum_{n_B^2=1}^{n_B^1} P(n_B^2, t) \dots \sum_{n_B^k}^{n_B^{k-1}} P(n_B^k, t) P(n_B - n_B^k, t). \tag{24}$$

We found that we could predict the clone size distributions of Lrig1CREER stripes by taking $\lambda = 3$ and using otherwise the same maximum likelihood parameters as for K14 cells. This suggests that the clonal dynamics of K14 expressing cells might be preceded burst of proliferation in Lrig1 expressing cells. However, we need to exercise some caution in the interpretation of these results: while we expect the merging rate of these clones to be only slightly higher than for K14CREER clones, abundant merging could potentially explain the differences between K14CREER and Lrig1CREER clones equally well.

In summary, clonal analysis suggests that K14 expressing cells that form stripes into the wound largely recapitulate the clonal dynamics of clonal dynamics in homeostasis. This dynamics is, up to a time independent convolution, maintained for Lrig1CREER stripes originating from hair follicles.

Supplementary References

1. Mascre, G. *et al.* Distinct contribution of stem and progenitor cells to epidermal maintenance. *Nature* **489**, 257–262 (2012).

2. Klein, A. M. & Simons, B. D. Universal patterns of stem cell fate in cycling adult tissues. *Development* **138**, 3103–11 (2011).

3. Klein, A., Doupé, D., Jones, P. & Simons, B. Kinetics of cell division in epidermal maintenance. *Phys. Rev. E* **76**, 021910 (2007).

4. Watson, J. K. *et al.* Clonal dynamics reveal two distinct populations of basal cells in slow-turnover airway epithelium. *Cell Reports* **12**, 90–101 (2015).

5. Box, G. E. P. & Tiao, G. C. *Bayesian inference in statistical analysis*, vol. 40 (John Wiley & Sons, 2011).

6. Gillespie, D. T. Exact stochastic simulation of coupled chemical reactions. *J. Phys. Chem.* **81**, 2340–2361 (1977).

7. Clayton, E. *et al.* A single type of progenitor cell maintains normal epidermis. *Nature* **446**, 185–189 (2007).

8. Bailey, N. T. J. *The Elements of Stochastic Processes with Applications to the Natural Sciences* (John Wiley & Sons, 1990).

9. Sánchez-Danés, A. *et al.* Defining the clonal dynamics leading to mouse skin tumour initiation. *Nature* DOI 10.1038/nature19069 (2016).
